# The *Sox2* regulatory region 42 produces enhancer RNAs that influence neuronal migration during cortical development

**DOI:** 10.1016/j.isci.2026.117332

**Published:** 2026-08-26

**Authors:** Satoe Banno, Tsukasa Sanosaka, Ryo Tomooka, Noriko Mizota, Akinori Tokunaga, Hideyuki Okano, Jun Kohyama

**Affiliations:** 1 Department of Physiology, Keio University School of Medicine, Shinjuku-ku, Tokyo 160-8582, Japan; 2 Laboratory of Stem Cell Biology, Faculty of Human Sciences, Waseda University, Tokorozawa, Saitama 359-1192, Japan; 3 Division of Laboratory Animal Resources, Life Science Research Laboratory, University of Fukui, Eiheiji, Fukui 910-1193, Japan; 4 Keio University Regenerative Medicine Research Center, 3-25-10 Tonomachi, Kawasaki-ku, Kawasaki, Kanagawa 210-0821, Japan; 5 Department of Orthopedic Surgery, Keio University School of Medicine, Shinjuku-ku, Tokyo 160-8582, Japan

**Keywords:** mouse, cortex, neural stem cells, neuronal migration, snRNA-seq, snATAC-seq, enhancer, eRNAs

## Abstract

Precise regulation of neural progenitor states and neuronal positioning is essential for cortical development. Here, we identified Sox2 regulatory region 42 (SRR42), a regulatory region located 42 kb downstream of the Sox2 transcription start site that exhibits open chromatin, active histone modifications, and bidirectional transcription of enhancer RNAs (eRNAs). Using transgenic reporter mice, we found that SRR42 is active in neural progenitor cells in the developing cortex. CRISPR-Cas9-mediated deletion of SRR42 combined with single-cell multiome analysis revealed alterations in progenitor-state dynamics, neuronal migration, and cortical lamination without detectable changes in *Sox2* expression. Furthermore, *in vivo* knockdown of SRR42-derived eRNAs impaired neuronal migration. These findings identify SRR42-derived RNAs as regulators of neuronal migration during cortical development and highlight enhancer-associated transcription as a functional component of neurodevelopmental gene regulation.

## Highlights

• SRR42 is an active regulatory region in the developing cerebral cortex
• SRR42 activity is enriched in neural progenitor cells during corticogenesis
• SRR42 deletion disrupts neuronal migration and cortical lamination in mice
• SRR42-derived eRNAs regulate neuronal positioning during corticogenesis

## Graphical abstract

**Figure:**
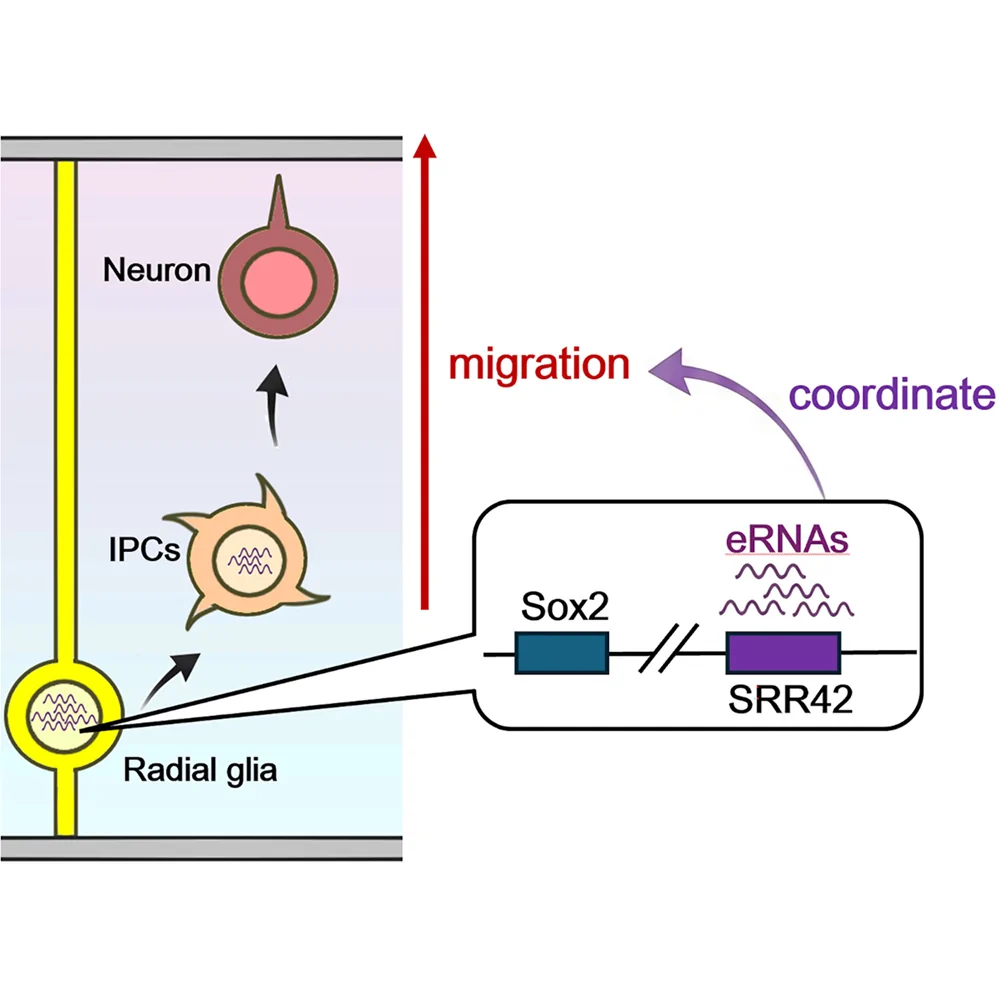

## Teaser

Molecular neuroscience; Developmental neuroscience; Cellular neuroscience

## Introduction

Enhancers are pivotal *cis*-regulatory elements that orchestrate spatiotemporal gene expression and play a crucial role in developmental processes across metazoan species.^1^^,^^2^ Genome-wide analyses have revealed that these elements are abundant, often cell type-specific, and shape unique transcriptional programs central to cellular identity.^3^^,^^4^^,^^5^ Transcription from enhancers has recently been demonstrated to be a prevalent phenomenon, which results in the production of enhancer RNAs (eRNAs), including those derived from clusters of potent composite enhancers known as super-enhancers (SEs), which frequently determine cell identity during developmental processes and in diseases such as cancer.^6^^,^^7^^,^^8^ Although eRNAs were initially thought to primarily function in *cis* to regulate the expression of nearby genes, their broader functional effects on developmental processes remain less understood, underscoring a critical gap in our comprehension of enhancer biology.

The mammalian cerebral cortex serves as an excellent model for dissecting gene regulatory mechanisms underlying complex developmental processes. The proper generation and positioning of neurons in the cortex depend on tightly regulated transitions within neural progenitor cells (NPCs).^9^^,^^10^^,^^11^ A specialized subset of NPCs known as radial glia (RG) in the ventricular zone (VZ) undergo self-renewal during early neural development, and subsequently generate intermediate progenitor cells (IPCs) in the subventricular zone (SVZ), which produce neurons in the upper layer of the cortex.^12^^,^^13^ These fate transitions are governed by dynamic gene expression programs, many of which are orchestrated by enhancer activity.^14^^,^^15^ Misregulation of such programs can result in impaired neurogenesis, disrupted lamination, and cortical malformations, as seen in several neurodevelopmental disorders.

Among key transcription factors in neural development, Sox2 plays an essential role in regulating various types of stem cells and tissue progenitors,^16^^,^^17^^,^^18^^,^^19^^,^^20^ including NPCs. *Sox2* expression is controlled by more than 20 distinct enhancers^21^^,^^22^ located in a 1.5 Mb gene desert, some of which function as distal super-enhancers that shape the expression of *Sox2*.^23^^,^^24^ Previous studies have defined multiple Sox2 regulatory regions (SRRs) according to their genomic distance from the Sox2 transcription start site (TSS). For example, SRR18 is located 18 kb upstream, whereas SRR107 and SRR111 are positioned 107 and 111 kb downstream of the TSS, respectively. Importantly, SRR107 and SRR111 have been shown to be essential for Sox2 regulation in embryonic stem (ES) cells.^24^ This distance-based nomenclature has been widely adopted to ensure clarity and consistency across studies of Sox2 regulation.^24^^,^^25^^,^^26^ Additionally, the *Sox2* locus harbors a long non-coding RNA gene, termed *Sox2* overlapping transcript (*Sox2ot*), which is co-expressed with *Sox2* and implicated in both human lung and breast cancer formation^27^^,^^28^ and in regulating *Sox2* expression.^29^ Consequently, elucidating the transcriptional regulatory mechanisms of *Sox2* is essential for understanding both developmental processes and tumorigenesis. However, the contribution of individual distal regulatory elements and their associated non-coding transcripts to neurodevelopmental events such as neuronal migration remains largely unexplored, especially regarding their capacity to produce functional eRNAs during development.

In this study, we identified a conserved distal enhancer element located downstream of Sox2. In accordance with the established SRR nomenclature, and because this element resides approximately 42 kb downstream of the Sox2 TSS, we refer to it as Sox2 regulatory region 42 (SRR42). This enhancer exhibits hallmarks of activity, including open chromatin structure, histone modifications, and bidirectional transcription indicative of eRNA production, particularly in RG populations. To investigate the function of this element and its transcribed eRNAs, we used a multi-pronged approach combining transgenic reporter assays, CRISPR-Cas9-mediated deletion of the SRR42 locus, and Cas13-based knockdown of SRR42-derived eRNAs. Our findings revealed that SRR42 activity is required for the proper migration of neural progenitor-derived cells during cortical development, independently of *Sox2* expression changes. These results suggest that enhancer-derived RNAs may serve as critical regulators of neural progenitor behavior *in vivo*, expanding the functional repertoire of non-coding transcription in developmental gene regulatory networks.

## Results

### Identification of distal enhancer regions involved in the regulation of the *Sox2* gene

**Figure 1:**
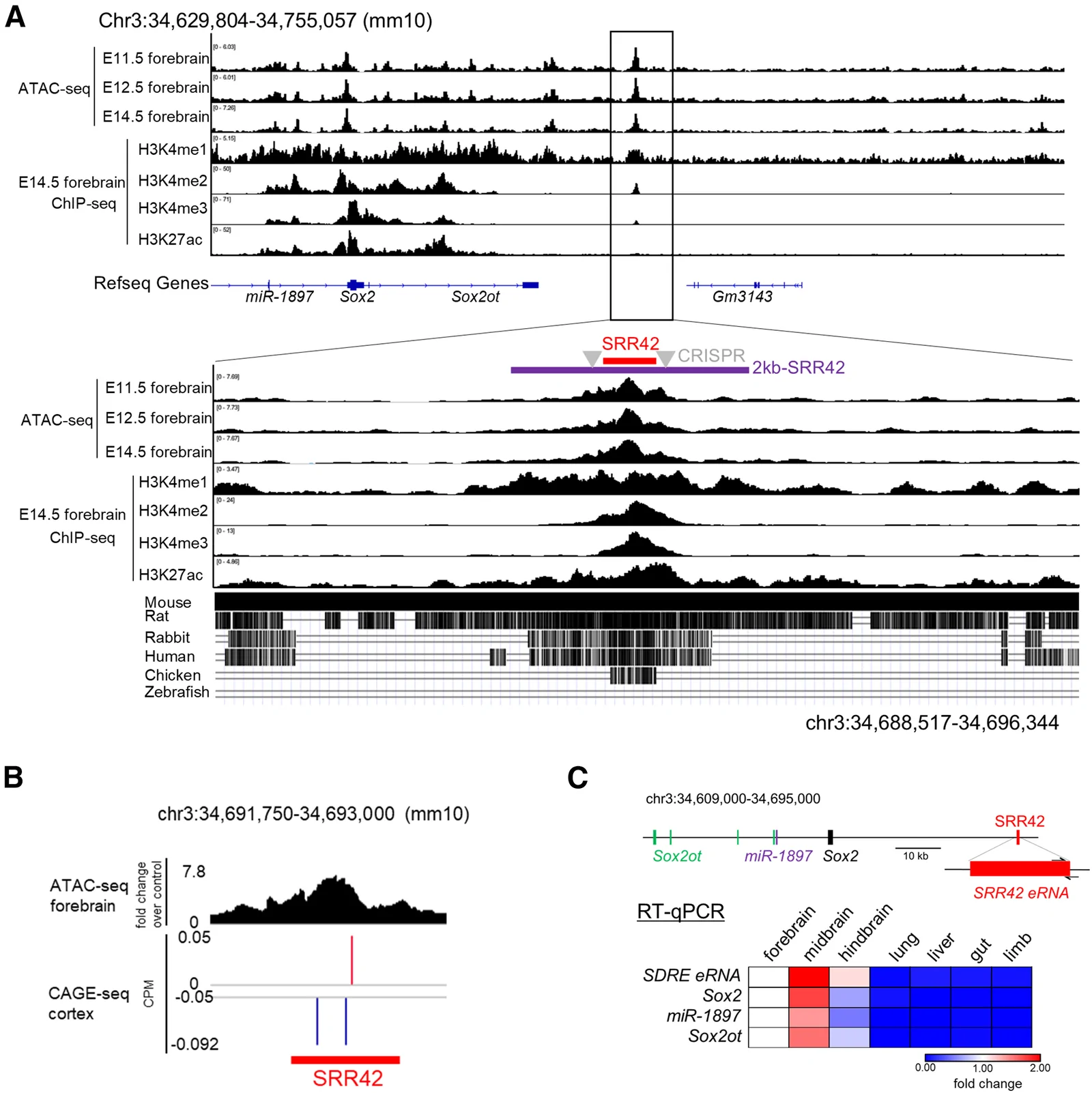
Identification of an active enhancer in the developing cortex of mice at E14.5 (A) Genome browser view of ATAC-seq signals in the forebrain regions at embryonic day 11.5 (E11.5), E12.5, and E14.5 together with chromatin immunoprecipitation sequencing (ChIP-seq) profiles for representative histone modifications at E14.5, focusing on the *Sox2* gene locus. The region boxed by a black rectangle corresponds to the Sox2 regulatory region 42 (SRR42) and is expanded to show evolutionary conservation across species. SRR42 is indicated by red lines, and CRISPR target sites for knockout mouse generation are shown by gray inverted triangles. The purple line represents the reporter sequence for transgenic mice. ATAC-seq and ChIP-seq datasets were obtained from the Encyclopedia of DNA Elements (ENCODE) project (https://www.encodeproject.org/). (B) Chromatin accessibility at the SRR42 region in the E14.5 mouse forebrain, shown with ENCODE ATAC-seq data and bidirectional transcription revealed by CAGE-seq. (C) Heatmap displaying the relative expression levels of SRR42 enhancer RNA (eRNA) and adjacent genes across tissues derived from E14.5. Expression levels were normalized to Gapdh and calculated relative to the forebrain sample, which was set to 1. Heatmap colors represent log2-transformed relative expression values. An enlarged schematic of the SRR42 region illustrates the location and orientation of the RT-qPCR primers used to quantify SRR42 eRNA expression. The sequences of the primers used are listed in Table S1.

The *Sox2* locus is surrounded by multiple distal regulatory elements; however, the specific role of these enhancers during neural development remains incompletely understood.^22^^,^^23^^,^^30^^,^^31^^,^^32^ To identify active enhancer candidates, we analyzed the public assay for transposase-accessible chromatin using sequencing (ATAC-seq) database derived from the embryonic mouse forebrain.^33^^,^^34^^,^^35^^,^^36^^,^^37^ As shown in Figure 1A, ATAC-seq profiles at embryonic days 11.5 (E11.5), 12.5 (E12.5), and 14.5 (E14.5) revealed several regions of open chromatin in the vicinity of *Sox2*. Among these regions, we identified a particularly intriguing region located 42 kb downstream of *Sox2*, which exhibits consistent accessibility across developmental stages. This region, termed SRR42, also showed conservation across species and enrichment of enhancer marks such as H3K4me1 and H3K27ac, and notably H3K4me3, which correlates with transcriptional activity^38^^,^^39^ (Figure 1A). To compare SRR42 with previously characterized SRRs, we examined chromatin accessibility and histone modification profiles at SRR18, SRR85, SRR95, SRR107, and SRR111 (Figure S1). This analysis showed that SRRs exhibit distinct activity-associated chromatin features depending on developmental stage and cellular context. SRR42 displayed stronger enhancer-associated chromatin features in the E14.5 forebrain than in mES cells, suggesting that SRR42 is active in a developmental context distinct from several previously characterized SRRs.

Recent studies have demonstrated that eRNAs are synthesized from enhancer regions and function in both *cis*- and *trans*-regulatory manners.^7^^,^^40^^,^^41^ Additionally, eRNA transcription occurs bidirectionally from enhancer regions and can be detected using cap analysis of gene expression (CAGE)-seq.^42^^,^^43^^,^^44^ Consequently, to assess transcriptional activity at the SRR42 locus, we performed CAGE-seq using RNA from E14.5 mouse cortex. CAGE-seq revealed bidirectional transcription at the SRR42 site, a characteristic feature of eRNAs (Figure 1B). Expression of SRR42-derived transcripts was further examined across embryonic tissues. We also analyzed the expression of *Sox2* and its neighboring genes including *miR-1897* and *Sox2ot* (Figure 1C). As shown in Figure 1C, SRR42 eRNAs were preferentially expressed in neural tissues, mirroring the expression patterns of neighboring genes including *Sox2*, *Sox2ot*, and *miR-1897*.

We next investigated whether the SRR42 region is similarly active in human neural lineages. Single-cell ATAC-seq data from the CATlas^45^ for human brain development demonstrated open chromatin at the SRR42-equivalent locus in several human RG populations, including Rgl_Tel_1, Rgl_Tel_2, and Rgl_Tel_dorsal (Figure S2A). CAGE-seq of human induced pluripotent stem cells (hiPSC)-derived neurospheres^46^ also showed bidirectional transcription at the SRR42-equivalent locus (Figure S2B), indicating that this regulatory region is conserved and transcriptionally active in both mouse and human neural progenitors.

### SRR42 enhancer activity in the developing cerebral cortex

**Figure 2:**
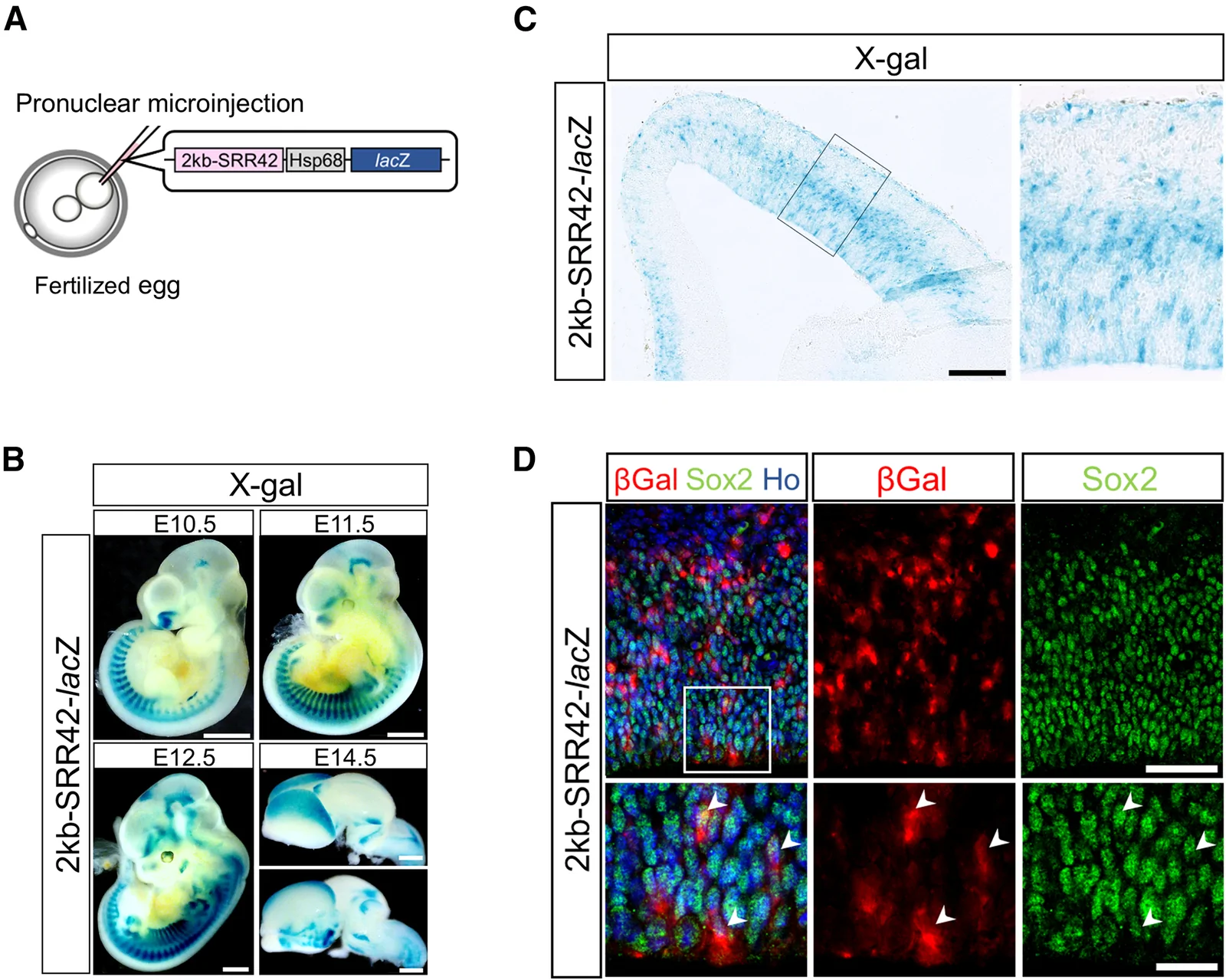
Reporter analysis of SRR42 enhancer activity in the embryonic cerebral cortex (A) Schematic representation of the generation of a transgenic mouse line using the SRR42-2 kb reporter construct. The 2 kb enhancer fragment (chr3:34,691,323–34,693,322) drives lacZ expression. (B) Reporter activity in E10.5, E11.5, and E12.5 embryos and E14.5 brain in the SRR42-2 kb reporter mice. X-gal staining was performed on line #1 at the indicated developmental stages. Scale bars, 1 mm. (C) Representative X-gal staining in the E14.5 cortex. The boxed area on the left is shown at a higher magnification in the right image. Scale bar, 100 μm. (D) Representative image of immunohistochemistry against β-Gal (red), Sox2 (green), and Hoechst (blue) in the E14.5 cortex. Arrowheads indicate cells co-expressing Sox2 and β-Gal. Scale bars, 50 μm. Antibody sources and dilutions, along with Hoechst staining details, are provided in Table S2.

In the cerebral cortex of E14.5 mice, transcription predicted to represent eRNAs from the SRR42 was observed; however, it remained unclear whether the SRR42 possesses enhancer activity. To verify the enhancer activity of the SRR42, we generated SRR42 reporter mice and spatially investigated whether the SRR42 functions as an enhancer. Specifically, we generated transgenic mice by connecting a minimal mouse heat shock promoter and a *lacZ* reporter gene^47^ to a 2 kb genomic fragment encompassing the SRR42 sequence (chr3:34,691,323–34,693,322) (SRR42-2 kb; Figure 2A). Among eight founder lines, three (#1, #2, and #3) exhibited X-gal staining indicative of reporter activation (Figure S3). Line #1, which showed the strongest and most consistent staining, was selected for further analysis.

To assess the developmental expression pattern of the SRR42-2 kb *lac*Z reporter, embryos from line #1 were collected at E10.5, E11.5, E12.5, and E14.5 and subjected to whole-mount X-gal staining. As shown in Figure 2B, at E10.5, *lacZ* expression was first detected in the olfactory placode, neural tube, and dorsal root ganglia. Similar X-gal staining results were observed in other E10.5 embryos, confirming the reproducibility of our findings (Figure S4). This expression continued to increase with time, and by E14.5, reporter activity was prominently observed in the dorsal forebrain (Figure 2B). Sectional analysis of the E14.5 cortex revealed strong X-gal staining in the VZ, suggesting localized enhancer activity in neural progenitor regions (Figure 2C). The X-gal staining results revealed that the SRR42 functions in neural tissues, including but not limited to regions of Sox2 expression, such as the olfactory placode, neural tube, and cortex. Immunohistochemical analysis using an antibody against β-galactosidase confirmed that β-galactosidase expression overlapped with Sox2-positive neural stem cells (NSCs) in the VZ (Figure 2D). Additionally, a subset of β-Gal-positive cells lacking Sox2 expression was observed. To further characterize the cellular context of SRR42 reporter activity, we performed co-staining with Neurogenin2 and Tbr2, markers associated with neuronal commitment and intermediate progenitor states, respectively (Figure S5). LacZ-positive cells in the apical region showed limited overlap with Neurogenin2 or Tbr2, whereas partial overlap was observed in more basally located LacZ-positive cells.

These findings indicate that the SRR42 acts as an active enhancer in the embryonic cortex, particularly in Sox2-expressing NSCs within the VZ. The spatial and temporal specificity of its activity suggests that SRR42 contributes to the transcriptional regulation of the neural progenitor identity during cortical development.

### Generation and phenotyping analysis of *SRR42*-knockout mice

To clarify the *in vivo* function of SRR42, we generated knockout (KO) mice using the CRISPR-Cas9 system via electroporation.^48^ CRISPR-Cas9 components, including guide RNA (gRNA) targeting sequences upstream and downstream of the SRR42 locus and Cas9 protein, were prepared and introduced into pronuclear-stage embryos (Figures S6A and S6B). The oocytes were cultured until the two-cell stage and subsequently transferred to pseudopregnant female mice. Genotyping by PCR and sequencing confirmed the deletion of the SRR42 region in four of five founder animals (Figures S6B and S6C). Two independent lines (#3 and #5) carrying complete SRR42 deletions were used for subsequent analysis.

**Figure 3:**
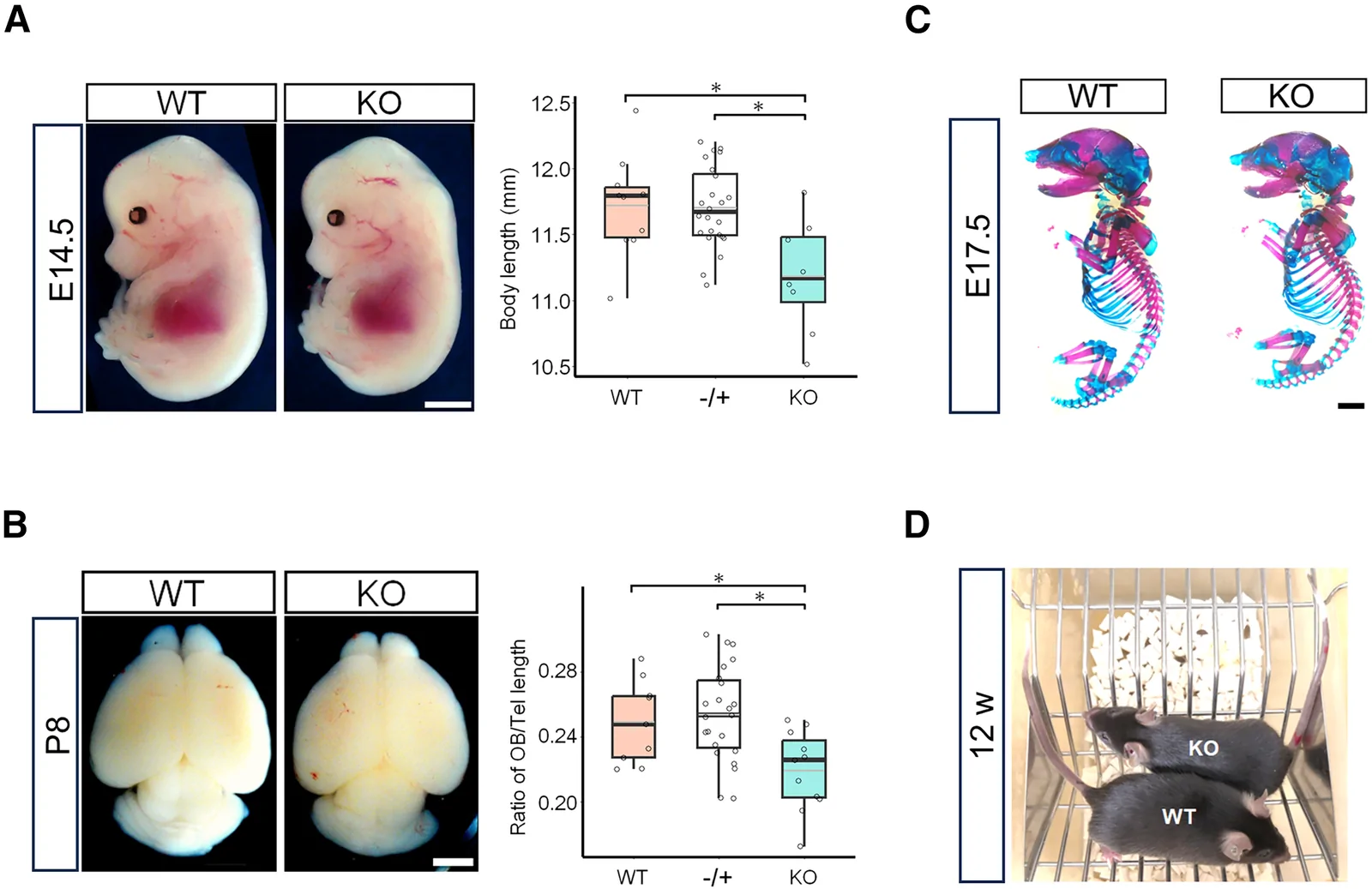
Generation of *SRR42*-knockout (KO) mice by electroporation of CRISPR-Cas9 complex (A) Representative images of E14.5 wild type (WT) and *SRR42*-KO embryos. Scale bar, 2 mm. The graph on the right shows a quantitative comparison of body length between WT and KO embryos. Each data point is shown as an open circle. The black line represents the median, while the gray line indicates the mean. ∗*p* < 0.05, Tukey-Kramer test (*n* = 10 WT, 24 +/−, 8 KO mice). (B) Representative images of postnatal day 8 (P8) brains of WT and KO mice. Scale bar, 2 mm. The graph on the right shows the ratio of olfactory bulb length to cerebrum length. Each data point is shown as an open circle. The black line represents the median, while the gray line indicates the mean. ∗*p* < 0.05, Tukey-Kramer test (*n* = 9 WT, 23 +/−, 11 KO mice). (C) Sagittal view of E17.5 WT and KO embryos stained with alizarin red (bone, red) and alcian blue (cartilage, blue). Scale bar, 2 mm. (D) Gross appearance of adult WT and KO mice. KO mice were viable and exhibited normal growth comparable to WT littermates.

At E14.5, KO embryos appeared grossly normal, but they exhibited a modest yet statistically significant reduction in body length compared with wild type (WT) and heterozygous littermates (Figures 3A and S6D). At postnatal day 8 (P8), the ratio of olfactory bulb (OB) length to telencephalon length^49^ was significantly lower in KO mice than in WT and heterozygous mice (Figure 3B), indicating potential alterations in the brain development. To further characterize the OB phenotype, we examined apoptosis and progenitor proliferation in the P0 OB. Quantification of Cleaved-Caspase3-positive cells and phospho-histone 3 (PH3)/Tbr2 double-positive progenitors^50^^,^^51^ revealed no significant differences between WT and SRR42-KO mice (Figures S7A and S7B). In addition, SRR42 reporter activity was detected in the P0 OB (Figures S7C and S7D). These findings suggest that SRR42 deletion affects early brain structures without causing gross morphological abnormalities or major changes in apoptosis or progenitor proliferation at birth. In contrast, skeletal staining of E17.5 embryos revealed no overt differences between WT and KO animals (Figure 3C), and adult KO mice were viable and exhibited normal external morphology and growth patterns (Figure 3D).

These findings suggest that SRR42 deletion affects early brain structures without causing gross morphological abnormalities.

### Single-cell multiomics analysis reveals chromatin and transcriptional alterations in *SRR42*-knockout cortex

**Figure 4:**
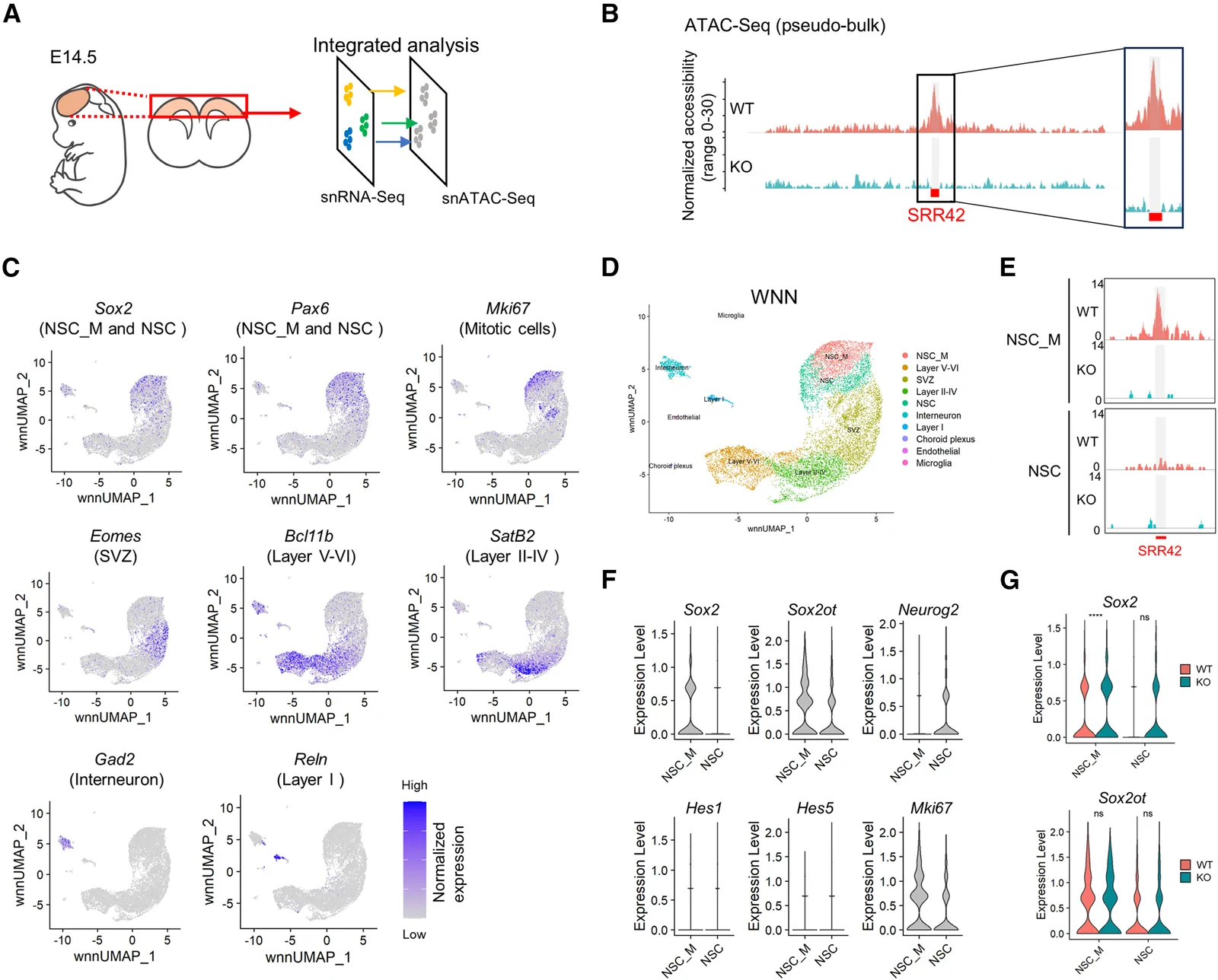
Single-cell multiome analysis of snRNA-seq and snATAC-seq in *SRR42*-KO and WT cortices (A) Schematic overview of single nuclear RNA-seq (snRNA-seq) and ATAC-seq (snATAC-seq) performed on embryonic cortical tissues from WT and KO mice. (B) Chromatin accessibility profiles around the SRR42 locus (chr3:34,682,107–34,702,549; mm10), shown as pseudo-bulk tracks derived from pooled snATAC-seq data. Red and green signals represent WT and KO samples, respectively. The SRR42 region is indicated by a red line and shown at a higher magnification on the right. (C) A set of UMAP projections showing the scaled expression of marker genes for distinct cortical cell populations: *Sox2* (NSC_M and NSC), *Pax6* (NSC_M and NSC), *Mki67* (mitotic cells), *Eomes* (SVZ), *Bcl11b* (layers V–VI), *SatB2* (layers II–IV), *Gad2* (interneurons), and *Reln* (layer I). High expression regions are indicated in blue, while low expression regions are indicated in gray. (D) UMAP projection of integrated weighted nearest neighbor (WNN) analysis, colored by annotated cortical cell types. (E) Chromatin accessibility profiles around the SRR42 locus (chr3:34,687,107–34,697,549; mm10), based on pseudo-bulk snATAC-seq data from NSC_M and NSC subsets. (F) Violin plot displaying the expression of *Sox2*, *Sox2ot*, *Neurog2*, *Hes1*, *Hes5*, and *Mki67* in NSC_M and NSC populations. (G) Violin plot showing the expression of *Sox2* and *Sox2ot* in NSC_M (WT: *n* = 942, KO: *n* = 882 cells) and NSC (WT: *n* = 818, KO: *n* = 694 cells) populations. ∗∗∗∗*p* < 0.0001; ns, not significant; Welch’s *t* test.

To explore the cellular and molecular consequences of SRR42 deletion, we performed a combined analysis using single-nucleus RNA sequencing (snRNA-seq) and single-nuclear snATAC-seq^52^^,^^53^^,^^54^^,^^55^ on E14.5 cortical tissues from WT and KO embryos (Figure 4A). This integrated single-cell multiomics approach enabled simultaneous profiling of chromatin accessibility and gene expression across thousands of individual nuclei. This approach enabled direct comparison of transcriptional states and chromatin accessibility within the same individual cells, allowing identification of cell type-specific regulatory features associated with SRR42 deletion. In total, 6,222 nuclei from WT and 5,242 nuclei from KO cortices were retained after quality CTRL filtering (Figure S8A). Quality metrics including RNA counts, TSS enrichment, nucleosome signal, and mitochondrial read content were comparable between genotypes (Figure S8B).

Pseudo-bulk analysis of the snATAC-seq data revealed a loss of chromatin accessibility at the SRR42 locus in KO samples, confirming the genomic deletion (Figure 4B).

Next, uniform manifold approximation and projection (UMAP) was created by dimensionality reduction of the snRNA-seq and snATAC-seq data, followed by weighted nearest neighbor (WNN) analysis,^52^ representing a weighted combination of RNA and ATAC-seq modalities (Figure S8C). This analysis revealed that our datasets include 17 transcriptionally and epigenetically distinct clusters (Figures S8C, S9A, and S9B). These clusters were further classified into nine major cell types based on known marker genes, referencing cortical cell-types annotation frameworks,^54^^,^^56^ including NSCs (NSC_M and NSC; *Sox2*, *Pax6*), mitotic cells (NSC_M; *Mki67*), intermediate progenitors (SVZ; *Eomes*), projection neurons (layers V–VI and layers II–IV; *Bcl11b* and *SatB2*, respectively), interneurons (*Gad2*), layer I neurons (*Reln*), microglia (*Trem2*), endothelial cells (*Igfbp7*), and choroid plexus cells (*Otx2*) (Figures 4C, 4D, S9B, and S9C). It is worth noting that we subdivided the NSCs cluster into two groups: NSC and NSC_M. The NSC_M population corresponds to mitotically active NSCs characterized by high Mki67 expression, consistent with previous report.^44^ To validate the consistency between RNA-based cell type annotations and chromatin accessibility profiles, we performed multimodal label transfer analysis^55^ between the single-cell RNA sequencing (scRNA-seq) and scATAC-seq modalities (Figure S10). This analysis showed concordance between transcriptome-based and ATAC-based cell identity assignments, supporting the reliability of the integrated single-cell multiome-based cell-type annotation. To further characterize progenitor heterogeneity, we performed trajectory analysis using Monocle3^57^ based on pseudotime reconstruction (Figure S11A). This analysis positioned NSC_M cells at the earliest pseudotime states, suggesting that this population represents a relatively undifferentiated progenitor state compared with the broader NSC population.

To determine which cell types have active chromatin at the SRR42 locus, we analyzed the pseudo-bulk snATAC-seq profiles across the identified cell types. Chromatin accessibility at the SRR42 locus was highest in neural stem/progenitor populations, particularly NSC_M and NSC, with additional substantially weaker signals in SVZ progenitors and upper-layer projection neurons (Figure S9D). This pattern closely parallels prior reporter activity, suggesting cell type-specific enhancer function of SRR42.

To further resolve enhancer usage between closely related populations, we compared chromatin accessibility at the SRR42 region between NSC_M and NSC populations. Notably, the NSC_M population exhibited a distinct and robust peak at the SRR42 locus, whereas the NSC population showed only weak accessibility (Figure 4E). These findings indicate that SRR42 enhancer activity is especially prominent in the NSC_M population.

We next assessed gene expression profiles within the NSC_M and NSC populations. *Sox2* expression was predominantly enriched in the NSC_M population, while both NSC_M and NSC populations expressed *Sox2ot* and *Mki67*, consistent with proliferative activity (Figure 4F). *Neurog2* was expressed in NSCs, suggesting neuronal commitment in these progenitor pools.

To investigate the effect of SRR42 deletion on local transcriptional programs, we analyzed the expression of genes neighboring the SRR42 locus. In KO samples, Sox2 expression was significantly increased in NSC_M cells compared with NSCs, whereas Sox2ot expression was largely unaffected (Figure 4G). To determine whether the observed upregulation of Sox2 mRNA resulted in a corresponding increase in protein levels, we performed quantitative immunohistochemical analysis on E14.5 cortical sections from WT and SRR42-KO mice (Figure S12). We found that the overall proportion of Sox2-positive cells was significantly increased in the SRR42-KO cortex compared with the WT (Figures S12A and S12B, left). However, the mean fluorescence intensity—a measure of the protein level per individual cell—showed no significant difference between WT and KO (Figures S12A and S12B, right). Furthermore, we detected no significant difference in Sox2 protein abundance between E14.5 WT and KO cortices by Western blot analysis (Figure S12C). These results suggest that while the pool of Sox2-expressing cells expands in the absence of SRR42, the concentration of Sox2 protein within individual cells is maintained at a stable level, likely through robust post-transcriptional or post-translational regulatory mechanisms. To examine whether SRR42 deletion alters accessibility at other SRRs, we analyzed scATAC-seq profiles across previously characterized SRR loci (Figures S13A and S13B). Increased chromatin accessibility was observed at SRR18 specifically in SRR42-KO NSC_M cells, whereas major changes were not detected at several other known SRR regions. Because SRR18 is a known neural stem/progenitor enhancer,^24^^,^^58^ these findings suggest that SRR42 deletion may alter the balance of enhancer activity within the Sox2 regulatory landscape.

The expression of standard RG markers, such as *Fabp7*, *Slc1a3*, and *Vim*, exhibited genotype-dependent variations, indicating that the effect may be influenced by cell type (Figure S9E). The number of differentially expressed genes (DEGs) varied across cell types, with the most pronounced shifts observed in the NSC_M, NSC, and SVZ populations (Figure S9F). *Gfap* and *Hes1* were not detectably expressed in either NSC_M or NSC populations. In contrast, *Hes5* expression was increased in both NSC_M and NSC populations in the KO cortex (Figure S9E), indicating possible changes in Notch signaling regulation. Together, these data demonstrate that the SRR42 is selectively active in the NSC_M population and that its deletion leads to targeted transcriptional changes in NSC lineages without disrupting general stem cell identity. The selective enrichment of SRR42 chromatin accessibility in the NSC_M population, along with its heightened transcriptional responsiveness to SRR42 loss, suggests that less committed NSCs are more sensitive to enhancer perturbations during early cortical development.

### SRR42 deletion impairs neural progenitor cell migration and cortical architecture

**Figure 5:**
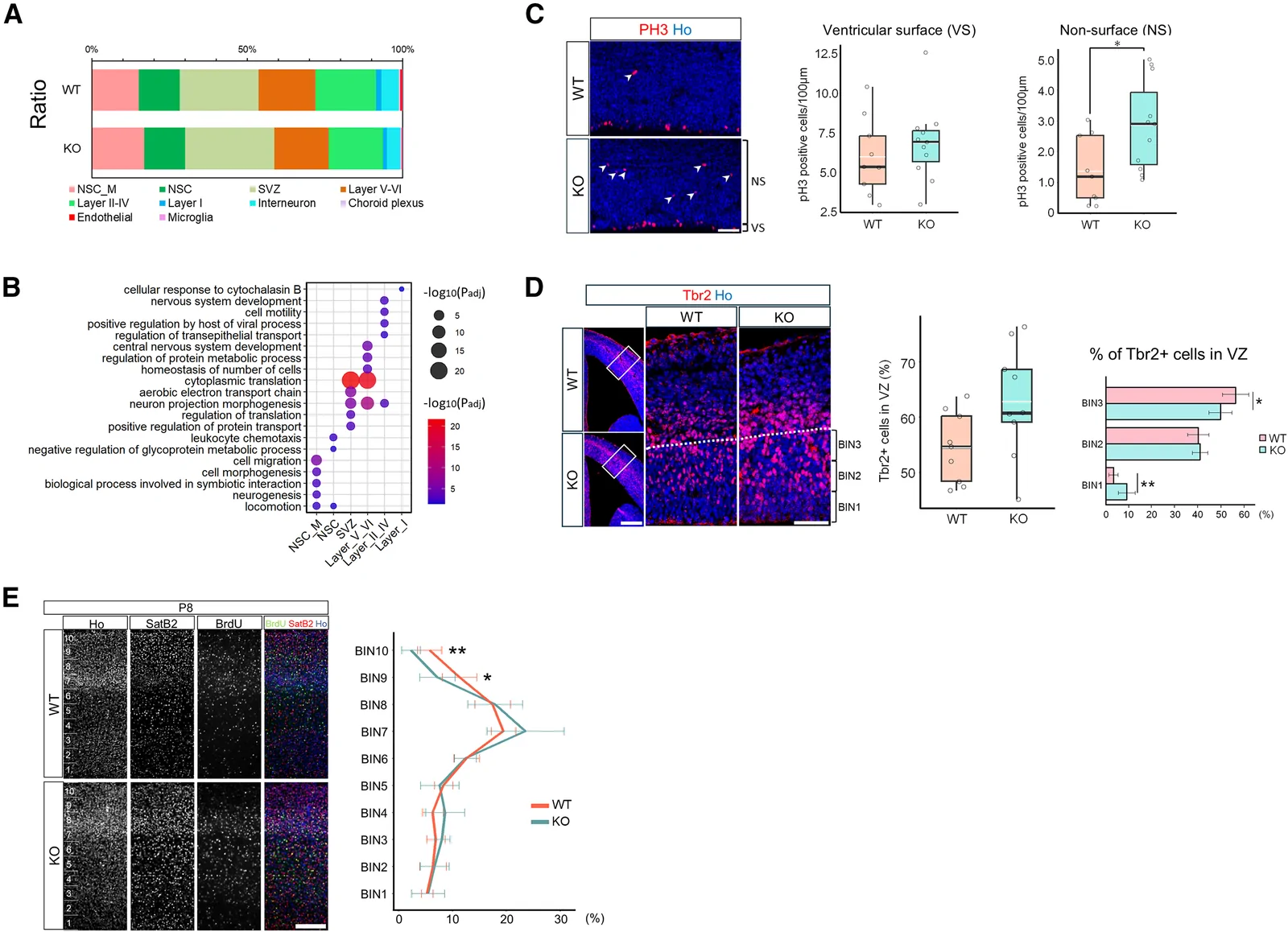
SRR42 regulates neural progenitor cell migration in the developing cerebral cortex (A) Horizontal stacked 100% bar chart displaying the proportion of each cortical cell type in WT and KO cortices. (B) Dot plot representing Gene Ontology (GO) enrichment analysis of differentially expressed genes (DEGs) between WT and KO samples across cortical cell types. GO terms related to biological processes are ranked by significance (–log_10_ adjusted *p* value). The analysis was performed using g:Profiler (https://biit.cs.ut.ee/gprofiler/gost). (C) Immunohistochemical staining of phosphorylated histone H3 (PH3) in E14.5 cortices of WT and KO mice, with PH3 labeled in red and nuclei counterstained blue with Hoechst. Scale bar, 50 μm. White arrowheads indicate NS-localized PH3^+^ cells. Quantification of PH3^+^ cells located along the ventricular surface (VS) or outside the surface (non-surface; NS) is shown on the right. Values represent the number of PH3^+^ cells per 100 μm of ventricular length. Each data point is shown as an open circle. The black line represents the median, while the white line indicates the mean. ∗*p* < 0.05, two-tailed unpaired Student’s *t* test (*n* = 9 sections from *N* = 3 mice per genotype). (D) Immunohistochemistry for Tbr2 (Eomes) in the E14.5 cortex of WT and KO mice, with Tbr2 shown in red and nuclei counterstained with Hoechst (blue). Scale bar, 50 μm. Left graph: percentage of Tbr2^+^ cells within the ventricular zone (VZ) relative to the total. Each data point is shown as an open circle. The black line represents the median, while the white line indicates the mean. Right graph: distribution of Tbr2^+^ cells within the VZ, subdivided into three equal bins (BIN1–BIN3). Data are presented as the mean ± standard deviation (SD). ∗*p* < 0.05; ∗∗*p* < 0.01, two-tailed unpaired Student’s *t* test (*n* = 9 sections from *N* = 3 mice per genotype). (E) BrdU was administered at E14.5, and P8 cortical sections were analyzed by immunohistochemistry for BrdU incorporation. Immunohistochemical staining shows BrdU in green, SatB2 in red, and nuclei counterstained with Hoechst in blue. Scale bar, 200 μm. Graphs displaying the distribution of BrdU^+^ cells across 10 equally spaced bins along the cortical depth from layer II to layer VI in WT and KO mice. Data are presented as the mean ± SD. ∗*p* < 0.05; ∗∗*p* < 0.01, two-tailed unpaired Student’s *t* test (*n* = 9 sections from *N* = 3 mice per genotype). The sources and dilutions of the antibodies used are listed in Table S2.

To assess the *in vivo* effect of SRR42 deletion on cortical development, we analyzed cellular composition, gene expression, and progenitor dynamics in the embryonic mouse brain. Cell-type composition analysis based on single-cell multimodal data revealed a slight increase in mitotically active NSC_M and SVZ populations in KO cortices, while the proportions of interneurons and projection neuron populations in layers II–IV and V–VI were slightly reduced compared with WT (Figure 5A). We also examined genotype-dependent differences along the differentiation trajectory (Figure S11B). KO cells were relatively enriched at early pseudotime states, whereas WT cells were more abundant at later differentiation states. These findings suggest altered progression of SRR42-KO progenitors along the differentiation trajectory. Because cell-type composition analysis revealed changes not only in NSC_M but also in SVZ and cortical layer populations, we examined the differences in gene expression in cortical cells between WT and KO mice (Figure S9F) and performed Gene Ontology (GO) analysis (Figure 5B). In the NSC_M population, DEGs were enriched in terms associated with cell migration. In the SVZ population, genes related to “neuron projection morphogenesis” were differentially expressed, while in layers II–IV and layers V–VI, DEGs were associated with central nervous system development (Figure 5B). Given the observed changes in genes related to neural development, we investigated cell division of neural progenitors by immunohistochemistry for PH3, a mitotic marker, in cortical sections^59^ at E14.5 (Figure 5C). While the number of PH3^+^ cells along the ventricular surface (VS) was comparable between WT and KO embryos, the KO cortex in regions outside the VS (referred to as non-surface [NS] in Figure 5C) exhibited a significant increase in the number of PH3^+^ cells, compared with WT mice, suggesting aberrant positioning of dividing cells (Figure 5C).

Following this analysis, we focused on the localization and distribution of specific progenitor cell populations. In particular, we examined the distribution of Tbr2 (Eomes)-positive cells, as their positioning within the cortical layers can provide further insight into the proliferative differences observed between WT and KO mice.^59^^,^^60^ At E14.5, we observed that the overall proportion of Tbr2^+^ cells in the VZ was modestly higher in KO mice than in the WT mice (Figure 5D). To gain more detailed insights, we subdivided the VZ into three compartments and analyzed the distribution of Tbr2^+^ cells within each compartment. Spatial analysis within the VZ revealed a significant accumulation of Tbr2^+^ cells in BIN1, the compartment adjacent to the VS, in KO mice. In contrast, Tbr2^+^ cells in BIN3, located near the SVZ, were more abundant in WT mice (Figure 5D). These findings suggest that progenitor positioning is altered in the absence of SRR42.

To evaluate whether these early defects in the progenitor positioning affect long-term cortical organization, we administered BrdU to pregnant mice at E14.5, and subsequently analyzed the cortex of the mice^61^ at P8 (Figure 5E). Compared with WT, KO cortices exhibited an altered distribution of BrdU^+^ cells across cortical layers, with a shift toward deeper positions, indicating delayed or disrupted migration (Figure 5E). Heterotopia is found in the cortex of mice with abnormal neuronal migration.^61^^,^^62^^,^^63^ Upon examination of the cortex of mice at P8, dysplasia was evident in the cortex of KO mice, including disorganized neuronal layering (Figure S15A) and abnormal Nestin-positive cellular aggregates in the cortical plate (Figure S15B). At E18.5, immunostaining for Satb2 and Ctip2 revealed the presence of heterotopic clusters of projection neurons, further confirming aberrant migration (Figure S15C). Together, these results demonstrate that SRR42 deletion disrupts progenitor positioning and migration during embryonic development, ultimately leading to cortical malformation.

### SRR42-derived eRNAs regulate neuronal migration in the embryonic cortex

**Figure 6:**
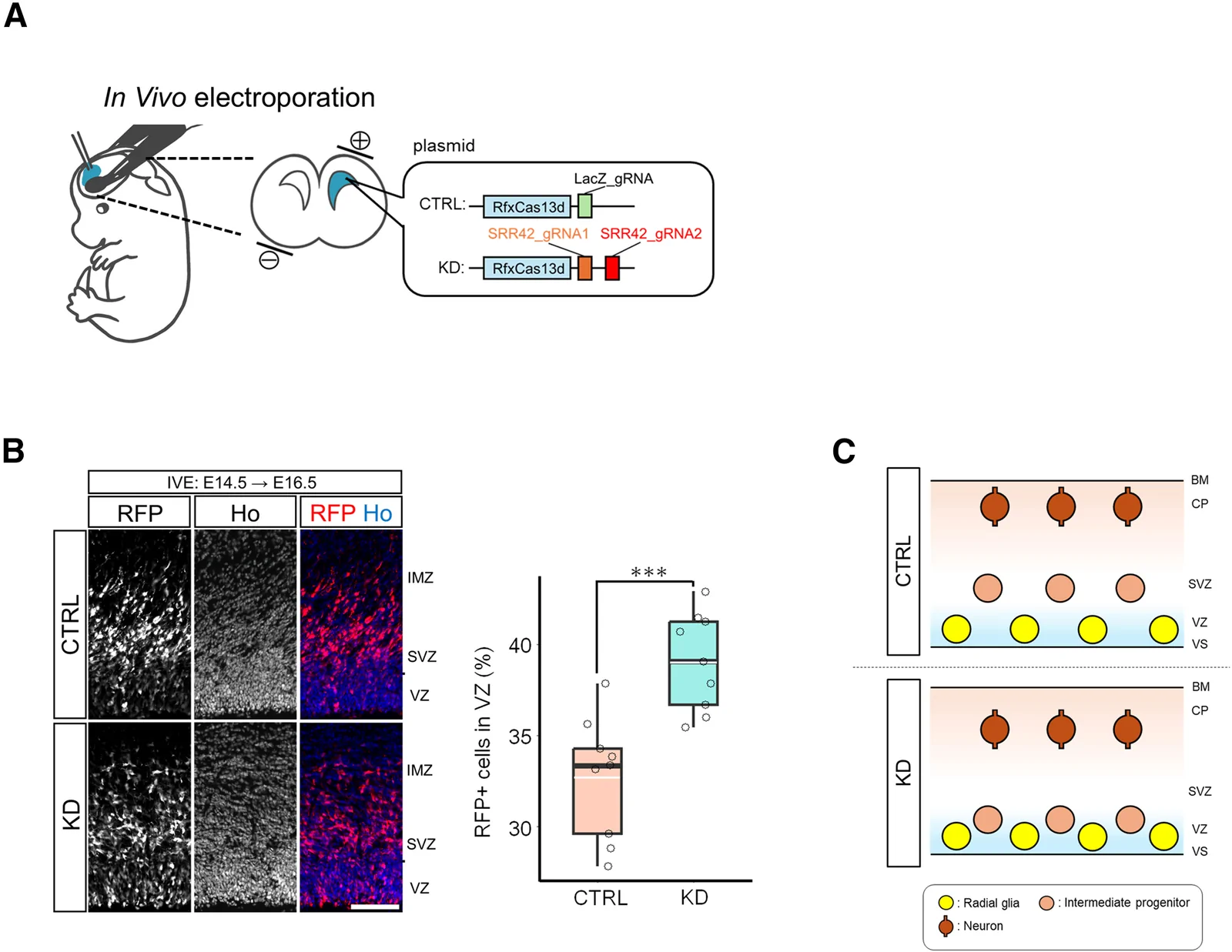
SRR42 eRNAs are involved in regulating neuronal cell migration (A) Schematic demonstration of the *in vivo* electroporation procedure. The tissue samples were collected 48 h after the electroporation. (B) Immunohistochemical analysis of neuronal migration in the E16.5 cortex following knockdown (KD) of SRR42 eRNAs. pCAGGS-RFP was co-electroporated as a fluorescent marker, with RFP shown in red and nuclei counterstained with Hoechst (blue). Scale bar, 100 μm. Cell migration was quantified by calculating the proportion of RFP-positive cells localized in the ventricular zone (VZ) relative to the total number of RFP-positive cells. Each data point is shown as an open circle. The black line represents the median, while the white line indicates the mean. ∗∗∗*p* < 0.001, two-tailed unpaired Student’s *t* test (*n* = 9 sections from *N* = 3 mice per group). The sources of the plasmids used, as well as the sources and dilutions of the antibodies, along with Hoechst staining details, are listed in Table S2. (C) Schematic representation of control (CTRL) versus knockdown (KD) cortical development. KD leads to the delayed migration of IPCs and neurons, with a higher density of IPCs remaining in the VZ compared with the control. Abbreviations: VZ, ventricular zone; SVZ, subventricular zone; IZ, intermediate zone; CP, cortical plate; BM, basement membrane; VS, ventricular surface.

To directly assess the functional role of SRR42-derived eRNAs in neuronal development, we used an RNA-targeting CRISPR system with RfxCas13d^64^^,^^65^^,^^66^ to knock down SRR42 eRNA transcripts. Accordingly, RfxCas13d and SRR42-specific gRNAs served as the knockdown (KD) vector, while *lac*Z-targeting gRNAs served as the negative CTRL. To confirm the efficacy of the Cas13d-mediated knockdown (KD), we quantified expression levels of SRR42 eRNA and neighboring transcripts, including Sox2 and miR-1897, in primary cortical cells *in vitro.* Reverse transcription-quantitative PCR (RT-qPCR) analysis revealed a significant reduction in SRR42 eRNA levels following KD (*p* < 0.05, Figure S14). In contrast, expression levels of Sox2 (*p* = 0.6857) and miR-1897 (*p* = 0.2026) were not significantly altered. These findings indicate that Cas13d-mediated KD reduces SRR42-derived transcripts without detectably affecting neighboring RNAs under these experimental conditions (Figure S14). We next introduced the KD vector into the cortex of E14.5 mice via *in vivo* electroporation. A pCAGGS-RFP plasmid was co-electroporated to label transfected cells, and brains were collected 48 h later at E16.5 for analysis (Figure 6A).

Immunohistochemical analysis revealed that under CTRL conditions, RFP^+^ cells had largely migrated beyond the VZ, populating the SVZ and intermediate zone. In contrast, KD of SRR42 eRNAs resulted in a pronounced accumulation of RFP^+^ cells within the VZ, suggesting impaired radial migration. Quantification confirmed the significant increase observed in the proportion of RFP^+^ cells retained in the VZ in the KD group, compared with controls (Figure 6B). These results demonstrate that SRR42-derived eRNAs contribute to the regulation of neuronal progenitor cell migration in the developing cortex (Figure 6C).

## Discussion

In this study, we identified a conserved distal enhancer element SRR42 downstream of the *Sox2* locus and demonstrated that it plays a critical role in regulating neuronal progenitor cell migration during cortical development. Our findings show that SRR42 activity is not only manifested by canonical enhancer features such as chromatin accessibility and histone modifications, but also by the production of bidirectionally transcribed eRNAs (Figure 1B). Using CRISPR-Cas9-mediated deletion and *in vivo* knockdown approaches (Figures 6A and S6A), we provide functional evidence that SRR42-derived eRNAs influence progenitor positioning independently of *Sox2* transcriptional activation, highlighting a previously unappreciated regulatory layer in neurodevelopment.

Previous studies have established that enhancer regions can drive gene expression by facilitating long-range chromatin interactions and modulating transcription factor recruitment.^67^ More recently, eRNAs have emerged as potential regulatory molecules that contribute to these processes.^68^ However, the *in vivo* functions of eRNAs, particularly in the context of complex developmental processes such as corticogenesis, remain poorly understood. Our results extend the functional repertoire of eRNAs by demonstrating that those transcribed from SRR42 play a non-redundant role in orchestrating radial migration of neural progenitors.

Notably, the genomic region identified here as SRR42 overlaps with the previously characterized D1 enhancer. In chick, D1 was reported to drive Sox2 expression in the cephalic ectoderm and otic placode during early development.^22^ More recently, Iida et al. demonstrated that the mouse D1 enhancer, located 40–50 kb downstream of Sox2, is activated in the neural tube and neural crest through cooperative binding of SOX2 and ZIC2.^32^ Interestingly, deletion of the mouse D1 enhancer did not produce overt developmental defects, suggesting functional redundancy among Sox2 regulatory elements. Thus, while the D1 region has primarily been studied in the context of Sox2 transcriptional activation and enhancer redundancy, our findings suggest that transcripts produced from the SRR42/D1-overlapping region may additionally contribute to progenitor behavior during cortical development. These observations expand the potential functional significance of this locus beyond canonical enhancer-mediated transcriptional activation.

Our reporter analysis and single-cell multiomics profiling revealed that SRR42 activity is enriched in the NSC_M population during early corticogenesis, which exhibits the highest chromatin accessibility at the SRR42 locus (Figures 2 and 4E). Although the transgenic reporter construct contains the SRR42 sequence itself, we did not observe obvious alterations in endogenous Sox2 expression patterns or cortical morphology in SRR42-LacZ embryos, suggesting that the reporter construct does not grossly perturb Sox2 regulation under these conditions. The stronger accessibility of SRR42 in NSC_M compared with the broader NSC population may reflect cell-state-specific chromatin organization within the Sox2-associated regulatory landscape. Previous studies have shown that the Sox2 locus undergoes dynamic remodeling of chromatin architecture during the transition from ES cells to neural stem/progenitor cells, including changes in sub-TAD organization and enhancer-promoter interactions.^69^^,^^70^ In addition, SOX2 has been reported to bind regulatory regions within the Sox2-associated chromatin domain in NPCs. Given that NSC_M cells represent an early/proliferative progenitor state, SRR42 accessibility may be enhanced in this population through cell-state-specific chromatin configuration or transcription factor occupancy. Future chromatin interaction analyses will be required to directly test this possibility.

Interestingly, despite the successful deletion of the SRR42 locus in *SRR42*-KO mice, *Sox2* expression was upregulated in the NSC_M population (Figure 4G). SRR42 deletion was associated with increased chromatin accessibility at SRR18 in NSC_M cells. Because SRR18 is an established neural stem/progenitor enhancer,^24^^,^^58^ this observation raises the possibility that SRR42 contributes indirectly to Sox2-associated regulation through modulation of regulatory interactions among Sox2-associated regulatory regions rather than acting solely as a direct Sox2 enhancer itself. The relatively modest effect on Sox2ot expression further suggests that SRR42 does not globally regulate the entire Sox2-associated chromatin domain, but instead participates in selective regulatory interactions within the locus. These findings suggest that SRR42 may regulate distinct targets in a cell type-specific manner, and that its role in progenitor behavior may not be limited to canonical gene activation. Notably, *in vivo* knockdown of SRR42 eRNAs via Cas13d-mediated RNA targeting led to delayed neuronal migration (Figure 6), further supporting the idea that enhancer transcripts can directly influence cellular positioning during development.

Furthermore, GO analysis of *SRR42*-KO cells indicated that it may affect the migration of the NSC_M population, a proliferative subpopulation of NSCs marked by high Mki67 expression (Figure 5B). Additionally, immunohistochemical analyses demonstrated the accumulation of mitotic (PH3^+^; Figure 5C) cells, likely corresponding to NSC_M populations, and intermediate progenitor (Tbr2^+^; Figure 5D) cells outside the VS in SRR42-deficient cortices, as well as delayed migration of BrdU-labeled cells in postnatal brains (Figure 5E). These phenotypes were recapitulated by eRNA-specific knockdown, supporting a functional role for SRR42-derived eRNAs in regulating the dynamic behavior of progenitor cells during cortical layering (Figure 6B). Our findings raise important questions regarding the mechanisms by which SRR42-derived eRNAs regulate neuronal migration. Although the present study does not directly distinguish between *cis*- and *trans*-acting functions, several plausible models can be considered. The enrichment of chromatin accessibility and active histone marks at SRR42 is consistent with a *cis*-regulatory role involving local chromatin interactions. Conversely, the detection of SRR42-derived transcripts raises the possibility that these eRNAs function in trans through interactions with RNA-binding proteins. Although we examined representative neuronal migration- and cytoskeleton-associated genes, we did not observe a consistent transcriptional signature that clearly explains the migration phenotype. Therefore, the mechanism by which SRR42-derived eRNAs regulate neuronal migration remains unresolved. One possibility is that these eRNAs act through local chromatin interactions or RNA-protein interactions that influence progenitor behavior without causing large changes in steady-state expression of canonical cytoskeletal genes. Future studies using RNA interactome analysis, chromatin conformation assays, and live imaging will be required to distinguish these possibilities. In contrast to enhancer-associated transcripts at the Dlx1/2 locus,^71^^,^^72^^,^^73^ which are primarily linked to interneuron specification in the ventral forebrain, SRR42-derived eRNAs appear to function within cortical progenitor populations. This distinction raises the possibility that SRR42 contributes not to neuronal fate determination itself, but rather to progenitor behaviors associated with neuronal migration. Interestingly, SRR42 deficiency resulted in heterotopia-like abnormalities rather than widespread lamination reversal as observed in Reeler mutants.^74^^,^^75^ Because lamination reversal is typically associated with disruption of positional signaling pathways, whereas heterotopia may arise from impaired migratory progression or persistence, these findings suggest that SRR42-associated regulation may preferentially affect migration-related cellular programs. Although the precise mechanisms remain unresolved, pathways related to cytoskeletal organization or cell adhesion may contribute to these phenotypes.

Multimodal analysis enabled us to integrate the information on open chromatin states and gene expression patterns (Figures 4 and S9) to determine the state of individual cells. However, these analyses do not include spatial information about the cells. Recently, spatial transcriptomics enabled the spatial characterization of individual cells within tissue sections based on specific gene expression.^76^ Given that *SRR42*-KO mice exhibit delayed neuronal migration, spatial transcriptomics analysis of KO brain sections may help elucidate the mechanisms of neuronal migration by tracing the characteristics of individual cells.

In the *SRR42*-KO mice, the proportion of the OB was reduced at P8 (Figure 3B). Given the region-specific activity of the SRR42 enhancer as demonstrated in our reporter mouse analysis (Figure 2B), it is possible that the observed OB phenotype reflects a regionally autonomous effect of SRR42 deletion. The enhancer’s activity in the developing olfactory placode and neural tube suggests that SRR42 may exert direct regulatory CTRL over progenitor populations specific to the olfactory region. However, further experiments will be required to definitively determine whether the phenotype arises in a cell-autonomous manner within olfactory progenitors or is secondarily influenced by broader developmental disruptions. Moreover, although the reduction in OB size in SRR42-KO mice was relatively mild, this phenotype is consistent with the effect of perturbing a single regulatory element rather than a core developmental regulator.^77^^,^^78^ Complete loss of key factors involved in OB development can result in severe malformations,^79^^,^^80^ whereas regulatory element perturbations may produce more restricted or stage-specific effects. In SRR42-KO mice, apoptosis and progenitor proliferation in the P0 OB were not significantly altered, while SRR42 reporter activity was detected within the OB (Figures S7C and S7D). These findings suggest that the reduced OB size observed at P8 may reflect defects in postnatal maturation or refinement rather than major early defects in OB neurogenesis.^81^^,^^82^^,^^83^ Additionally, data from transgenic mice indicated that the SRR42 region is active not only during mid-fetal neurogenesis but also in specific parts of the adult brain (Figure S3). Although *SRR42*-KO mice are viable as adults, they may exhibit behavioral abnormalities. Behavioral analysis of these mice may further elucidate the overall function of SRR42.

Despite these insights, the precise molecular mechanisms by which SRR42 eRNAs exert their effects remain unclear. Potential mechanisms include modulation of transcriptional activity at specific loci, chromatin remodeling, and interactions with RNA-binding proteins that influence cytoskeletal dynamics or signaling pathways related to migration. Future work integrating spatial transcriptomics, RNA interactome profiling, and live-cell imaging will be essential to uncover how enhancer transcription is linked to progenitor behavior at the molecular level. In addition, because the transgenic reporter construct contains additional copies of the SRR42 sequence, a potential influence on endogenous regulatory states cannot be completely excluded, although no obvious gross abnormalities in cortical morphology or tissue organization were observed in SRR42-LacZ embryos.

In conclusion, our study identified SRR42 as a critical regulator of neuronal migration during cortical development through an eRNA-mediated mechanism. These findings advance our understanding of epigenetic regulation of cortical development and may have implications for neurodevelopmental disorders. Further investigation of enhancer elements, such as SRR42, will likely yield additional insights into the complex regulatory mechanisms governing brain development.

### Limitations of the study

This study has several limitations. First, our phenotypic analyses focused primarily on embryonic and early postnatal stages, leaving the long-term impacts of SRR42 deficiency on adult brain function and behavior unexplored; thus, future behavioral and neural circuitry analyses in adult mice are required. Second, *in vivo* experiments relied entirely on mouse models, which may not fully recapitulate the complex spatiotemporal dynamics of human corticogenesis, necessitating future studies using human-derived systems such as brain organoids. Third, the precise molecular mechanism by which SRR42-derived eRNAs regulate progenitor cell migration remains unresolved. Integrating spatial transcriptomics, RNA interactome profiling, and live-cell imaging in future research will be essential to clarify this mechanism.

## Resource availability

### Lead contact

Further information and requests for resources and reagents should be directed to and will be fulfilled by the lead contact, Jun Kohyama (jkohyama@waseda.jp).

### Materials availability

The SRR42 reporter and KO mouse lines generated in this study will be made available by the lead contact upon reasonable request.

### Data and code availability

• The high-throughput data used in this study have been deposited in the Gene Expression Omnibus (GEO, https://www.ncbi.nlm.nih.gov/geo/). The single-cell multiome dataset is available under accession number GEO: GSE282390, the CAGE dataset for mouse cortex under accession number GEO: GSE296925, and the CAGE dataset for hiPSC-derived neurospheres under accession number GEO: GSE297056.
• This paper does not report original code.
• Any additional information required to reanalyze the data reported in this paper is available from the lead contact upon request.

## Acknowledgments

We thank the members of the Department of Physiology, Keio University School of Medicine for discussions. We are also grateful to the Collaborative Research Resources, Keio University School of Medicine, for providing their technical support. We thank Dr. Shingo Miyawaki at Gifu University for his technical advice on the generation of knockout mice. We also thank Dr. Osamu Nakajima at Yamagata University for generating the transgenic animals. We thank CiRA for providing hiPSCs. We thank Dr. Masaki Kakeyama along with his laboratory students (Waseda University) for their technical assistance with animal care. This study was supported by JSPS KAKENHI (grant nos. 19H03623 and 24K02571 to J.K.) and Waseda University’s Global Research Center (GRC) Support Program (project no. GRC-kyoten-05). We thank Michal Bell, PhD, from Edanz (https://jp.edanz.com/ac) for editing a draft of this manuscript.

## Author contributions

Conceptualization, S.B. and J.K.; methodology, S.B., T.S., R.T., N.M., and A.T.; investigation, S.B., T.S., and J.K.; writing the original draft, S.B., and J.K.; reviewing and editing, H.O.; funding acquisition, J.K.

## Declaration of interests

Hideyuki Okano is a compensated scientific consultant of San Bio, Co., Ltd. and K Pharma Inc.

## Declaration of generative AI and AI-assisted technologies in the writing process

During the preparation of this work, the authors used ChatGPT and Paperpal for grammar refinement and typographical error correction. The authors reviewed and edited the output as needed and take full responsibility for the content of the published article.

## STAR★Methods

### Key resources table

**Table:** 

REAGENT or RESOURCE	SOURCE	IDENTIFIER
**Antibodies**		

Anti-β-galactosidase	MBL	Cat# M203-3; RRID: AB_10697365
Anti-Sox2	R&D Systems	Cat# MAB2018; RRID: AB_2286684
Anti-SatB2	Abcam	Cat# ab92446; RRID: AB_10563678
Anti-BrdU	Abcam	Cat# ab6326; RRID: AB_305426
Anti-Ctip2	Abcam	Cat# ab18465; RRID: AB_2064130
Anti-NeuN	Millipore	Cat# MAB377; RRID: AB_2298772
Anti-Phospho-Histone H3 (pH3)	Cell Signaling Technology	Cat# 9706; RRID: AB_331535
Anti-Tbr2	Millipore	Cat# AB15894; RRID: AB_10615604
Anti-Ds-Red	Nittobo Medical	Cat# MSFR101410
Anti-Nestin	Millipore	Cat# MAB353; RRID: AB_94911
Anti-Cleaved Caspase-3	Cell Signaling Technology	Cat# 9661; RRID: AB_2341188
Anti-Neurogenin-2	Millipore	Cat# MABN1814
Anti-GAPDH	Cell Signaling Technology	Cat# 2118; RRID: AB_561053

**Chemicals, peptides, and recombinant proteins**		

DMEM/F12 medium	Fujifilm Wako	Cat# 042-30795
B-27 Supplement (50×)	Gibco/Thermo Fisher Scientific	Cat# 17504-044
GlutaMAX (100×)	Gibco/Thermo Fisher Scientific	Cat# 35050-061
HBSS	Fujifilm Wako	Cat# 084-08345
Phosphate Buffered Saline(10×) (pH 7.4)	Nacalai Tesque	Cat# 27575-31
TrypLE Select	Gibco/Thermo Fisher Scientific	Cat# 12563-029
Bovine Fibronectin	Sigma-Aldrich	Cat# F4759
L-Ornithine homopolymer hydrobromide	Sigma-Aldrich	Cat# P3655
Water sterile-filtered	Sigma-Aldrich	Cat# W3500
Blocking One	Nacalai Tesque	Cat# 03953-66
4%-Paraformaldehyde Phosphate Buffer Solution	Nacalai Tesque	Cat# 09154-85
iFluor™ 555 Styramide	AAT Bioquest	Cat# 45027
ezDNase™ Enzyme	Thermo Fisher Scientific	Cat# 11766051
TRI Reagent	Sigma-Aldrich	Cat# T9424
Hoechst 33258	Invitrogen	Cat# H3569

**Critical commercial assays**		

Primary Mouse Neuron Nucleofector™ Kit	Lonza	Cat# VPG-1001
mirVana™ miRNA Isolation Kit	Thermo Fisher Scientific	Cat# AM1560
RNeasy Plus Mini Kit	QIAGEN	Cat# 74134
ReverTra Ace® qPCR RT Master Mix	TOYOBO	Cat# FSQ-201
Fast SYBR™ Green Master Mix	Thermo Fisher Scientific	Cat# 4385617
Chromium Next GEM Single Cell Multiome ATAC + Gene Expression Reagent Bundle (4 rxns)	10× Genomics	Cat# PN-1000285

**Deposited data**		

ATAC-seq_mouse (ENCODE)	The ENCODE Project Consortium et al.^33^	https://www.encodeproject.org/
ATAC-seq_human (CatLas)	Li et al.^45^	https://catlas.org/catlas/
ChIP-seq (ENCODE)	The ENCODE Project Consortium et al.^33^	https://www.encodeproject.org/
DNase-seq (ENCODE)	The ENCODE Project Consortium et al.^33^	https://www.encodeproject.org/
CAGE-seq_mouse E14.5 cortex	This study	GEO: GSE296925
CAGE-seq_hNSCs	This study	GEO: GSE297056
scATAC+scRNA-seq	This study	GEO: GSE282390

**Experimental models: Cell lines**		

hiPSC lines YZWJs513	CiRA Foundation	N/A
Primary mouse cortical neurons	This paper (Isolated from E14.5 mice)	N/A

**Experimental models: Organisms/strains**		

Mouse C57BL/6JJmsSlc	Japan SLC	RRID: MGI:5488963
Mouse Slc:ICR	Japan SLC	RRID: MGI:5462094
Mouse: SRR42-KO	This paper	N/A
Mouse: SRR42-2 kb lacZ reporter	This paper	N/A

**Oligonucleotides**		

Primers for construction	FASMAC	Table S1
Primers for genotyping PCR	FASMAC	Table S1
Primers for RT-qPCR	FASMAC	Table S1
crRNA for deficient mice	Integrated DNA Technologies (IDT)	Table S1

**Recombinant DNA**		

Hsp68-LacZ-Gateway	Addgene	Plasmid #37843
pENTR/D-TOPO vector	Thermo Scientific	K240020SP
Hsp68-LacZ-SDRE_transgene_2k	This study	N/A
pCAGGS-mRFP	Kind gift from Dr. Ayako Y. Murayama (Keio Univ.)	N/A
pCAGGS-mEGFP	Kind gift from Dr. Ayako Y. Murayama (Keio Univ.)	N/A
AAV-EFS-NLS-CasRx-U6-DR-sgRNA_LacZ-DR	Addgene	Plasmid #154003
AAV-EFS-NLS-CasRx-U6-DR-sgRNA_SDRE_1-2-DR	This study	N/A

**Software and algorithms**		

Imaris (11.0)	Oxford Instruments	RRID: SCR_007370
ZEN microscopy software (v3.10)	Carl Zeiss	RRID: SCR_013672
Design and Analysis (2.8.0)	Thermo Fisher Scientific	RRID: SCR_026962
Compass for SW (6.3.0)	ProteinSimple (Bio-Techne)	https://www.bio-techne.com/
Integrative Genomics Viewer (IGV; v2.8.11)	Broad Institute	https://www.bio-techne.com/
Cell Ranger ARC (v2.0.0)	10× Genomics	https://support.10xgenomics.com/
R (v4.1.3, or v4.4.1)	R Foundation	RRID: SCR_001905; https://www.r-project.org/
Bioconductor	Bioconductor Project	RRID: SCR_006442; https://bioconductor.org/
Seurat (v4.0.3, or v5.2.1)	Satija Lab	RRID: SCR_016341; https://satijalab.org/seurat/
Signac (v1.0.3, or v1.14.0)	Stuart Lab	RRID:SCR_021158; https://stuartlab.org/signac/
Monocle 3 (v1.0.0)	Trapnell Lab	RRID: SCR_018685; https://cole-trapnell-lab.github.io/monocle3/
GenomicRanges	Stuart Lab	RRID: SCR_000025
BSgenome.Mmusculus.UCSC.mm10 (v1.4.3)	Bioconductor; UCSC	https://bioconductor.org/packages/BSgenome.Mmusculus.UCSC.mm10
EnsDb.Mmusculus.v79 (v2.99.0)	Bioconductor; Ensembl	https://bioconductor.org/packages/EnsDb.Mmusculus.v79

### Experimental model and study participant details

#### Animals

All experimental procedures were conducted in accordance with the guidelines approved by the Experimental Animal Care Committee of Keio University School of Medicine (17038 and 09091) and the Research Center for Molecular Genetics of Yamagata University School of Medicine (31–78), the Faculty of Human Sciences at Waseda University (A25-122), and the Life Science Research Laboratory at the University of Fukui (R03090). C57BL/6J and ICR mice were obtained from Japan SLC (Shizuoka, Japan). Animals were maintained on a 12-h light/dark cycle, with *ad libitum* access to food and water. Time pregnancies were generated by mating heterozygous males and females, and studies utilized embryonic mice at E10.5, E11.5, E12.5, E14.5, E16.5, E17.5 and E18.5, as well as postnatal mice at P0 and P8, without distinguishing their sex. Mice were euthanized by cervical dislocation. Anesthesia was induced either via inhalation of isoflurane or by intraperitoneal injection of a three-agent anesthetic mixture comprising medetomidine (0.75 mg/kg), midazolam (4 mg/kg), and butorphanol (5 mg/kg).

#### Cell cultures

Primary cortical neurons were prepared using cerebral cortices harvested from E14.5 embryonic mice of undetermined sex. The dissociated cells were cultured in DMEM/F12 medium supplemented with 2% B-27 Supplement and 1× GlutaMAX on poly-ornithine- and fibronectin-coated dishes. For human cell experiments, neurospheres derived from the human induced pluripotent stem cell (hiPSC) line YZWJs513^84^^,^^85^ were utilized. The hiPSC line was provided by the Center for iPS Cell Research and Application (CiRA) at Kyoto University and maintained through passages as previously described.^86^ No new hiPSC lines were established in this study. The hiPSC line was authenticated using STR profiling and confirmed to be mycoplasma-free by the supplier (CiRA).

### Method details

#### Plasmid construction

The enhancer region corresponding to the SRR42 region was amplified by PCR using genomic DNA extracted from C57BL/6J wild type (WT) mice as the template. The amplified fragment was then cloned into the pENTR/D-TOPO vector (Thermo Fisher Scientific, Waltham, MA, USA). Subsequently, the insert containing the SRR42 region was transferred to the Hsp68-*lacZ*-Gateway vector (#37843; Addgene, Watertown, MA, USA) to generate SRR42-2 kb *lac*Z reporter plasmid utilizing the LR recombination reaction (Thermo Fisher Scientific). For knockdown experiments targeting RNA derived from the SRR42 region, we used the plasmid vector AAV-EFS-NLS-CasRx-U6-DR-sgRNA_LacZ-DR (#154003; Addgene), which expresses *CasRx* and *LacZ* sgRNA. The sgRNA target sequences were selected using the siDirect algorithm (http://sidirect2.rnai.jp/). Detailed information of primers and gRNA sequences are provided in Table S1.

#### Generation of reporter and knockout (KO) mice

To generate SRR42-2 kb *lac*Z reporter mice, a *lacZ* reporter fragment was excised from the reporter plasmid by restriction enzyme digestion, and subsequently purified. The purified fragment was microinjected into the pronuclei of the fertilized C57/BL×DBA2 F1 (BF1) zygotes. Genotyping of transgenic mice was performed by PCR, and primer sequences are listed in Table S1.

To generate *SRR42*-KO mice, genome editing was performed on *in vitro* fertilized eggs from C57BL6/J mice using the Technique for Animal Knockout system by Electroporation (TAKE) method.^30^ The genome editing components, including crRNA, tracrRNA, and Cas9 protein, were purchased from Integrated DNA Technologies (IDT, Coralville, IA, USA). Target sites for the crRNA were designed using the CRISPOR algorithm (http://crispor.tefor.net/crispor.py), and the corresponding sequences are provided in Table S1. Among the four independently generated mutant lines, lines #3 and #5 were selected for further analysis. All KO mice used in phenotypic studies were subjected to at least four generations of backcrossing onto the C57BL/6J background. Owing to the similarity in phenotypes between lines #3 and #5 (Figure S3D), data from both lines were combined in subsequent analyses.

#### BrdU assays

To assess cell proliferation *in vivo*, 5-bromo-2-deoxyuridine (BrdU; 50 mg/kg, Nacalai Tesque, Kyoto, Japan) was intraperitoneally administered to pregnant female mice at E14.5, as previously described.^37^ Time pregnancies were generated by mating heterozygous *SRR42*-KO males and females. Brain samples were collected from litters containing both WT and mutant offspring. Three brains from each genotype were analyzed to ensure consistent sampling.

#### *In vivo* electroporation

To perform *in utero* gene knockdown, a DNA solution containing RfxCas13d knockdown vector (5 μg/μL) and pCAGGS-mRFP vector^87^^,^^88^ (5 μg/μL) for fluorescent labeling was prepared at a 1:4 volume ratio. The mixture was injected into the ventricles of embryonic C57BL/6J mice at E14.5 using a glass capillary (GD-1; NARISHIGE, Setagaya, Japan). Electroporation was conducted with platinum electrodes (CUY650P5; Nepa Gene, Ichikawa-City, Chiba, Japan) connected to a pulse generator (CUY21, Nepa Gene), delivering six cycles of 35 V electrical pulses, each lasting 50 ms with a 950 ms interpulse interval. Following electroporation, the uterus was repositioned within the abdominal cavity, and the incision was surgically sutured. The embryos were collected at E16.5 for further analysis.

#### *In vitro* electroporation

Cerebral cortices were harvested from E14.5 mice, and the cells were dissociated by pipetting and passed through a cell strainer. The cells were then electroporated with either an RfxCas13d knockdown vector or a control vector, along with pCAGGS-EGFP^87^^,^^88^ at a 9:1 ratio, using the Mouse Neuron Nucleofector Kit and a Nucleofector 2b device (Lonza, Basel, Switzerland). Following transfection, the cells were cultured for 48 h in DMEM/F12 medium (Fujifilm Wako, Osaka, Japan) supplemented with 2% B-27 Supplement and 1× GlutaMAX (both from Gibco/Thermo Fisher Scientific, Waltham, MA, USA) on poly-ornithine- and fibronectin-coated dishes. EGFP-positive cells were then sorted using an SH800 flow cytometer (Sony, Tokyo, Japan), with dead cells excluded via propidium iodide (PI) staining.

#### Whole-mount skeletal staining

E17.5 embryos were harvested and carefully dissected to remove skin, internal organs, and adipose tissue. Specimens were fixed in 95% ethanol at room temperature for 5 days, followed by immersion in acetone for 2 days. Staining was performed using a solution containing 0.3% Alcian blue 8 GS in 70% ethanol, 0.1% alizarin red S in 95% ethanol, acetic acid, and 70% ethanol (mixed in a ratio of 1:1:1:17). The staining process was conducted at 37°C for 3 days. After washing with distilled water, specimens were cleared in 1% KOH for 48 h, followed by sequential dehydration in 20%, 50%, and 80% glycerol solutions in 1% KOH for 24 h, at room temperature. Finally, specimens were preserved in 100% glycerol at room temperature for 20 days.

#### X-Gal staining

Embryos were fixed in ice-cold PBS containing 2% formaldehyde, 0.2% glutaraldehyde, and 0.01% NP-40. The fixation times were adjusted to the developmental stage: 10 min for E10.5, 20 min for E11.5, and 30 min for E12.5 and E14.5 embryos. The embryos were then permeabilized with PBS containing 0.05% Tween 20 for 15 min. X-Gal staining was performed using 1× X-Gal solution (AMSBIO, Abingdon, UK) at 37°C until optimal signal development was achieved. The reaction was terminated by immersion in PBS containing 4% paraformaldehyde (PFA). For clearing, E12.5 embryos were treated sequentially with 10%, 20%, 50%, and 80% glycerol in PBS (12 h each). For E14.5 embryos, brains were cryoprotected in 20% sucrose, embedded in OCT compound (Sakura Finetek, Torrance, CA, USA), and sectioned at 12 μm using a CM3050S cryostat (Leica Biosystems, Nussloch, Germany).

#### Immunohistochemistry

Brains were collected from E14.5, E16.5, and P8 mice and fixed in 4% PFA in PBS. Embryonic brains were fixed for 5 h at 4°C. P8 brains were fixed overnight. After cryoprotection in 20% sucrose in PBS at 4°C overnight, brains were embedded in OCT compound and sectioned at a thickness of 12 μm. For antigen retrieval, the sections were autoclaved in citrate buffer (Dako Target Retrieval Solution, Citrate pH 6; Agilent Technologies, Santa Clara, CA, USA) at 105°C for 15 min. The sections were permeabilized in Blocking One (Nacalai Tesque, Kyoto, Japan) containing 0.1% Triton X-100 for 1 h at room temperature. Primary antibodies were diluted in Blocking One and incubated overnight at 4°C. Secondary antibodies and Hoechst 33258 were then diluted in Blocking One and incubated for 1 h at room temperature. The β-Gal antibody staining was performed using the Power Styramide Signal Amplification (PSA) with iFluor® 555 Tyramide (AAT Bioquest, Pleasanton, CA, USA). The details of the antibodies used are provided in Table S2.

#### Immunoblot analysis using simple western system

For sample preparation, the cerebral cortex of E14.5 mice was lysed and homogenized in RIPA buffer I containing 50 mM Tris-HCl (pH 8.0), 150 mM NaCl, 1 mM EDTA, 0.1% SDS, 1% Triton X-100, 0.1% sodium deoxycholate, and a protease inhibitor cocktail (Nacalai Tesque). The lysates were subsequently disrupted using a Bioruptor (Diagenode, Denville, NJ, USA). We analyzed protein expression using the Simple Western system (ProteinSimple, San Jose, CA, USA), a non-gel-based, automated capillary Western blot-like substitute. The preparation of samples, antibodies, and reagents, as well as the experimental procedures, were performed according to the ProteinSimple user manual. Automated separation electrophoresis and chemiluminescence detection were executed by the instruments, and the resulting digital chemiluminescence images were analyzed using Compass software (ProteinSimple). For quantitative analysis, the peak area of Sox2 was normalized to that of Gapdh. Detailed information regarding the primary antibodies is summarized in Table S2.

#### RNA purification and reverse transcription-quantitative PCR (RT-qPCR) analysis

Total RNA was extracted using either the RNeasy Mini Kit (Qiagen, Hilden Germany) or TRI Reagent (Sigma–Aldrich, St Louis, MO, USA), or the mirVana miRNA Isolation Kit (Thermo Fisher Scientific), following the manufacturers’ protocols. cDNA was synthesized using the ReverTraAce reverse transcription kit (TOYOBO, Osaka, Japan) after treatment with ezDNase Enzyme (Thermo Fisher Scientific). qPCR was performed using Fast SYBR Green Master Mix (Thermo Fisher Scientific) on the ViiA7 Real-Time PCR System (Applied Biosystems, Waltham, MA, USA). To confirm the absence of genomic DNA contamination, non-reverse-transcribed samples were analyzed simultaneously by qPCR.

Primer sequences are listed in Table S1.

#### Single-cell multiome (snRNA-Seq and snATAC-Seq)

A single-cell Multiome ATAC + Gene Expression library (v1, 10× GENOMICS) was prepared from cortical tissues of E14.5 WT and KO littermate embryos. Isolation of nuclei and library preparation were performed according to the 10× Genomics protocol. Cell Ranger ARC (version 2.0.0, 10× GENOMICS) was used for demultiplexing, alignment, filtering, barcode counting, peak calling, and counting of RNA-Seq and ATAC-Seq reads. To minimize sex-based variability in the datasets, a mouse reference genome mm10 excluding XY chromosome information was prepared before running the Cell Ranger ARC. Weighted nearest neighbor (WNN) analysis^34^ was conducted using approximately 6,000 WT and 5,000 KO nuclei. The analysis workflow included the use of Seurat (v4.0.3 and v5.2.1) and Signac (v1.0.3 and v1.14.0) (https://satijalab.org/seurat/articles/weighted_nearest_neighbor_analysis). SCtransform was applied to normalize the RNA counts, and term frequency inverse document frequency (TF-IDF) was used to normalize the ATAC peaks. A WNN graph integrating both modalities were used for UMAP visualization and clustering. Differentially expressed gene (DEG) analysis was performed using the FindMarkers function in Seurat. The gene ontology (GO) enrichment analysis was conducted using the DEG data via g:Profiler (https://biit.cs.ut.ee/gprofiler/gost). The default statistical background provided by g:Profiler was used, corresponding to all annotated *Mus musculus* genes in the Ensembl database.

#### Integrated pseudotime and cell fate analysis

Pseudotime inference on the integrated object was performed using Monocle3.^57^ Raw RNA count matrices and cell metadata were used to construct a cellDataSet object, retaining cluster assignments and WNN UMAP coordinates directly from Seurat. To maintain a continuous topological structure, a single principal graph was constructed across all cells without partitioning. Pseudotime was calculated by defining a specific structural node (designated as Point 1 in Figure S11B) as the developmental root. To evaluate cell fate distribution between wild-type (WT) and knockout (KO) trajectories, we quantified the cellular occupancy at key principal graph nodes representing developmental transitions (Points 1–3). Specifically, cells localized within a radial distance of 0.8 UMAP units from each target node were counted and normalized to the total cell number of the respective genotype. This normalized frequency was used to compare the distribution of WT and KO cells along the inferred differentiation trajectory.

#### Multimodal label transfer

To estimate gene-level transcriptional activity from the ATAC data, gene activity scores were computed using the GeneActivity function in Seurat. These scores were incorporated into the Seurat object as a new ‘ACTIVITY’ assay and subsequently log-normalized using a scale factor of 10,000. To predict ATAC-based cell types from the existing RNA annotations, transfer anchors between the modalities were identified using the FindTransferAnchors function. This step utilized Canonical Correlation Analysis (CCA) for dimensionality reduction, setting the SCT-normalized RNA assay as the reference and the ‘ACTIVITY’ assay as the query. Cell type predictions were then generated using the TransferData function, providing the RNA-annotated clusters as the reference data and utilizing Latent Semantic Indexing (LSI) components (dimensions 2 to 30) for weight reduction. To evaluate the accuracy of these cross-modality cell type predictions, a confusion matrix was constructed comparing the annotated RNA cell types to the predicted ATAC cell types. For targeted analysis, the “NSC_M” and “NSC” clusters were combined into a single “NSC_M + NSC” group, and the data was calculated as column-wise proportions to reflect the fraction of predicted ATAC identities per RNA-annotated cluster. Finally, the analysis was restricted to a specific subset of relevant clusters (“NSC_M + NSC”, “SVZ”, “Layer V-VI”, “Layer II-IV”, and “Layer I”), and the resulting transfer accuracy matrix was visualized as a heatmap using ggplot2 and the viridis color scale.

#### Cap analysis of gene expression (CAGE)-Seq

Cortical tissues from three WT E14.5 embryos and neurospheres derived from human induced pluripotent stem cells (hiPSCs) YZWJs513,^85^^,^^86^ provided by the Center for iPS Cell Research and Application (CiRA) at Kyoto University, were used for CAGE. Neurospheres were obtained by performing eight passages using a previously reported method.^84^ RNA integrity (RIN >7.0) was confirmed using a Bioanalyzer (Agilent). CAGE library preparation, sequencing, mapping, and gene expression analysis were conducted by K.K. DNAFORM (Yokohama, Japan). The cDNA was synthesized from total RNA using random primers, and the 5′ cap structures were biotinylated. The biotinylated RNA/cDNAs were then selected by streptavidin beads. Following RNA digestion and adapter ligation, double-stranded cDNA libraries were constructed and sequenced on a NextSeq 2000 platform (Illumina, San Diego, CA, USA). The resulting reads were mapped to the mouse mm10 or human hg38 genome using STAR. CAGE-tagged data were clustered using the Paraclu algorithm with CAGEr.^42^ Clusters with counts per million (CPM) below 0.2 were discarded for mouse samples, while clusters with CPM below 0.1 were discarded for human samples.

#### Image analysis and quantification

Whole embryos and brains images of mice were captured using the LPE-06BK imaging system (SANWA SUPPLY, Okayama City, Japan). Thin-sliced specimens were imaged using a confocal microscope (LSM700, Carl Zeiss, Oberkochen, Germany), a fluorescence microscope (BZ-X810, KEYENCE, Osaka, Japan), or an image cytometer (CQ1, Yokogawa Electric Corporation, Tokyo, Japan). Images of the sliced specimens were acquired from three separate locations within each brain, using two or three brains collected from littermates. Cell death in the olfactory bulb (OB) was assessed in P0 tissue sections using an anti-Cleaved-Caspase3 antibody. The number of Cleaved-Caspase3-positive cells was quantified and normalized to the section area (expressed as cells/mm^2^). Similarly, the density of Tbr2+/PH3+ double-positive cells was determined by dividing the cell count by the analyzed area. To ensure comparability between WT and KO, sections were anatomically matched along the anteroposterior axis of the OB based on Hoechst counterstaining. For the BrdU assay, P8 mouse brains were collected after BrdU injection at E14.5. Cortical layers II–VI were divided into 10 equal-sized bins, with the bin on the layer IV side designated as BIN1. BrdU^+^ cells were counted in each bin and expressed as a percentage of the total. For PH3 staining, cell proliferation was quantified by counting the number of PH3^+^ cells either lining the ventricular surface in the dorsal cortex (referred to as “Ventricular surface [VS]”) or located outside the ventricular surface in the cerebral cortex (referred to as “Non-surface [NS]”). These counts were then normalized by the length of the ventricular surface. The results were averaged to calculate the density of PH3^+^ cells per μm of ventricular surface of the dorsal cortex. Localization analysis of Tbr2 for the comparison of WT and KO at E14.5 was calculated by dividing the Tbr2-positive cells present in the VZ by the total Tbr2-positive cells. Also, the VZ was divided into compartments 1–3 (BIN1-3), and the proportion of where Tbr2 occupies within the VZ was calculated. For knockdown analyses, sections exhibiting high populations of fluorescently labeled cells were selected. The number of labeled cells in the ventricular zone was then divided by the total number of labeled cells to determine the proportion of labeled cells. To analyze Sox2-expressing cells, we compared the proportion of Sox2-positive cells in the cortex and their individual expression levels between WT and KO. The proportion was determined by normalizing the number of Sox2-positive cells to the total Hoechst count. To quantify per-cell expression levels, images were captured using constant exposure and gain settings; total fluorescence intensity was measured, and cells were segmented by intensity to calculate the mean value in arbitrary units (A.U.) per cell. Cell death in the olfactory bulb (OB) was assessed in P0 tissue sections using an anti-Cleaved-Caspase3 antibody. The number of Cleaved-Caspase3-positive cells was quantified and normalized to the section area (expressed as cells/mm^2^). Similarly, the density of Tbr2+/PH3+ double-positive cells was determined by dividing the cell count by the analyzed area. To ensure comparability between WT and KO, sections were anatomically matched along the anteroposterior axis of the OB based on Hoechst counterstaining. The detection and intensity quantification of antibody-stained positive cells were performed using Imaris software (Oxford Instruments, Abingdon-on-Thames, UK). Specifically, the “Spots” function was employed to identify and designate positive cells, while the “Surface” function was used for the detailed analysis of signal intensity.

### Quantification and statistical analysis

In the box-and-whisker plot, the black line represents the median, while the gray or white line indicates the mean. The lower and upper boundaries of the box represent the 25th and 75th percentile data, respectively. The whiskers extend to the most extreme data points, excluding any outliers. Actual data values are plotted as circles. To control for potential litter-to-litter variation in the Simple Western data, a linear model (lm function in R) was used with genotype as the main factor and litter (mother) as a blocking factor. Two-tailed Student’s *t* test, Tukey–Kramer test, Welch’s *t* test, paired *t* test, and Type II ANOVA were used to analyze statistical significance. The level of significance is indicated by asterisks: ∗*p* < 0.05; ∗∗*p* < 0.01; ∗∗∗*p* < 0.001; ∗∗∗∗*p* < 0.0001.
