# Supplementary material for: The Sox2 regulatory region 42 produces enhancer RNAs that influence neuronal migration during cortical development
Source: iScience. 2026 Aug 26;29(9):117332. doi: 10.1016/j.isci.2026.117332 (PMC13544347; doi:10.1016/j.isci.2026.117332)
Supplement: Supplementary file 1 [file mmc1.pdf]

iScience, Volume *29*

Supplemental information

The *Sox2* regulatory region 42 produces enhancer RNAs that influence neuronal migration during cortical development

Satoe Banno, Tsukasa Sanosaka, Ryo Tomooka, Noriko Mizota, Akinori Tokunaga, Hideyuki Okano, and Jun Kohyama

Supplemental Figures and Supplemental Figure legends

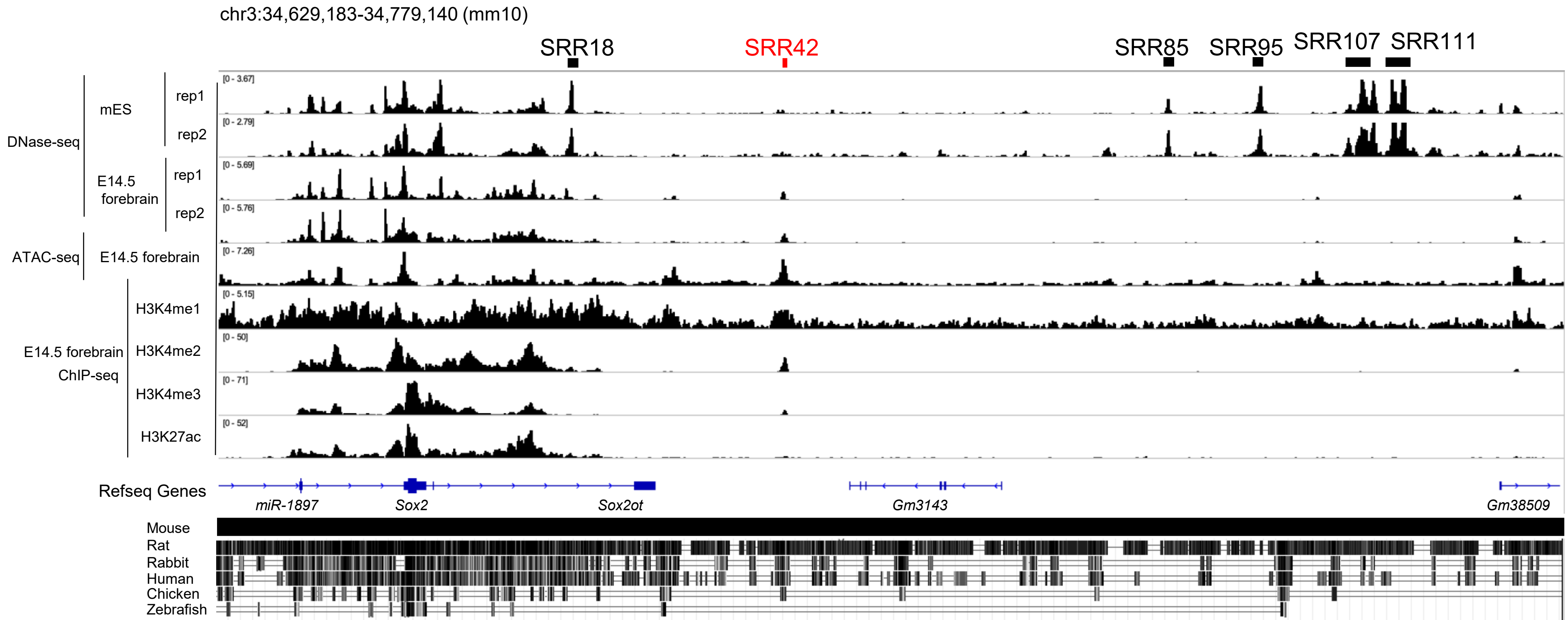
chr3:34,629,183-34,779,140 (mm10)
SRR18
SRR42
SRR85
SRR95
SRR107
SRR111
DNase-seq
mES
rep1
rep2
E14.5 forebrain
rep1
rep2
ATAC-seq
E14.5 forebrain
E14.5 forebrain ChIP-seq
H3K4me1
H3K4me2
H3K4me3
H3K27ac
Refseq Genes
miR-1897
Sox2
Sox2ot
Gm3143
Gm38509
Mouse
Rat
Rabbit
Human
Chicken
Zebrafish

Figure S1

Figure S1. Comparison of chromatin accessibility and histone modification profiles across Sox2 regulatory regions.
Genome browser views displaying chromatin accessibility (DNase-seq and ATAC-seq) and histone modification profiles (ChIP-seq) at the indicated SRR regions (SRR18, 42, 85, 95, 107, and 111) in mouse embryonic stem cells (mES) and E14.5 forebrain cells. Data tracks were obtained from the ENCODE project database.

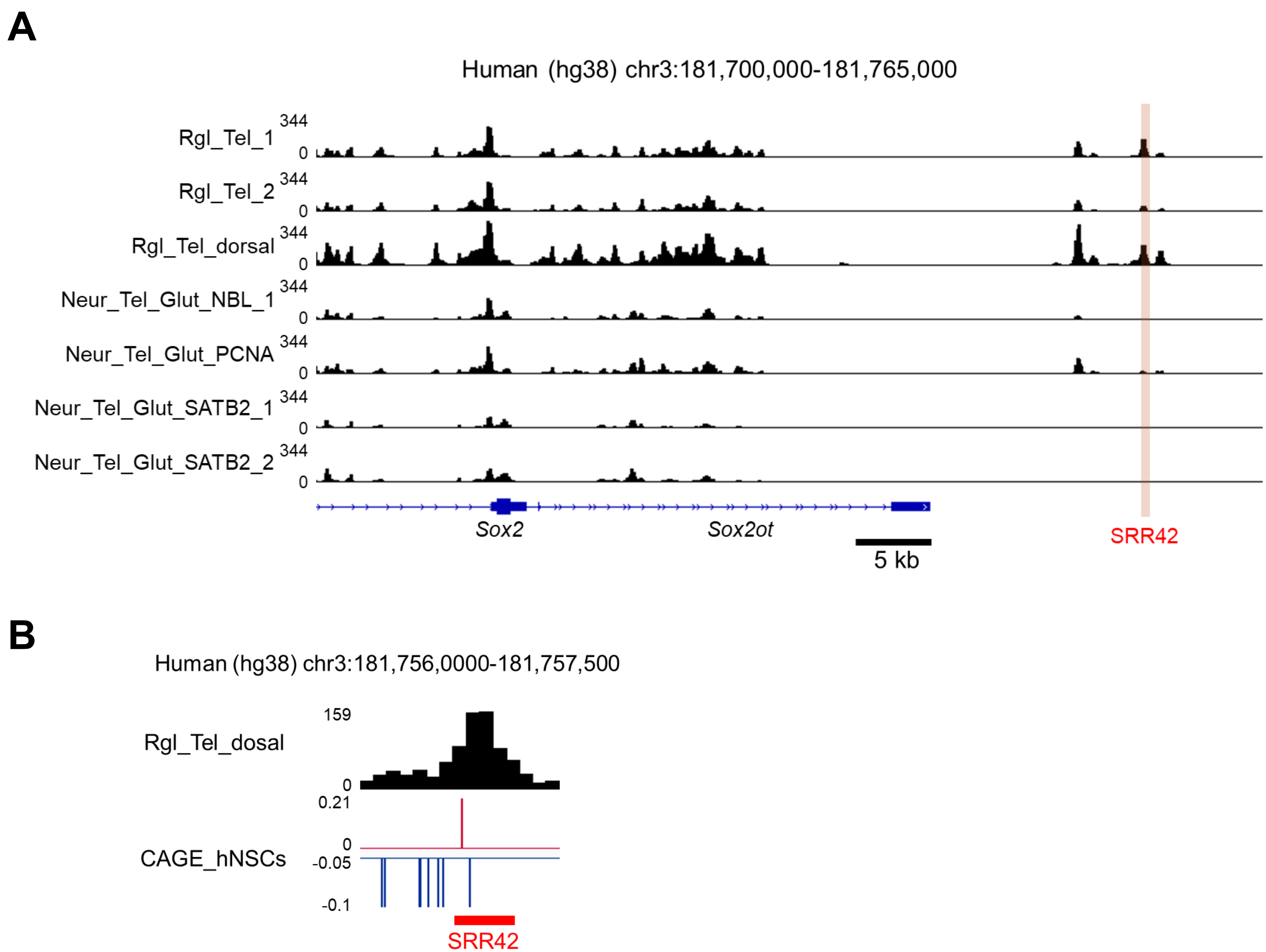
A
Human (hg38) chr3:181,700,000-181,765,000
Rgl_Tel_1
Rgl_Tel_2
Rgl_Tel_dorsal
Neur_Tel_Glut_NBL_1
Neur_Tel_Glut_PCNA
Neur_Tel_Glut_SATB2_1
Neur_Tel_Glut_SATB2_2
344
0
Sox2
Sox2ot
5 kb
SRR42
B
Human (hg38) chr3:181,756,0000-181,757,500
Rgl_Tel_dosal
159
0
0.21
0
-0.05
-0.1
CAGE_hNSCs
SRR42

Figure S2

Figure S2. Chromatin accessibility and transcriptional activity at the SRR42 equivalent region in human-derived NSCs.

A) The genome browser tracks demonstrating chromatin accessibility across representative human neural subtypes from snATAC-Seq at the indicated loci in the figure. The data were obtained from the CATlas for Human Brain Development of CATlas (http://catlas.org). Notably, radial glial cells (Rgl_Tel_1, Rgl_Tel_2, and Rgl_Tel_dorsal) exhibited accessibility at loci corresponding to the SRR42 region.

B) A genome browser visualization of chromatin accessibility in the SRR42 equivalent region in radial glia in the human dorsal telencephalon, as obtained from CATlas (http://catlas.org), together with bidirectional transcription at the region revealed by CAGE-Seq of human iPSC-derived neurospheres.

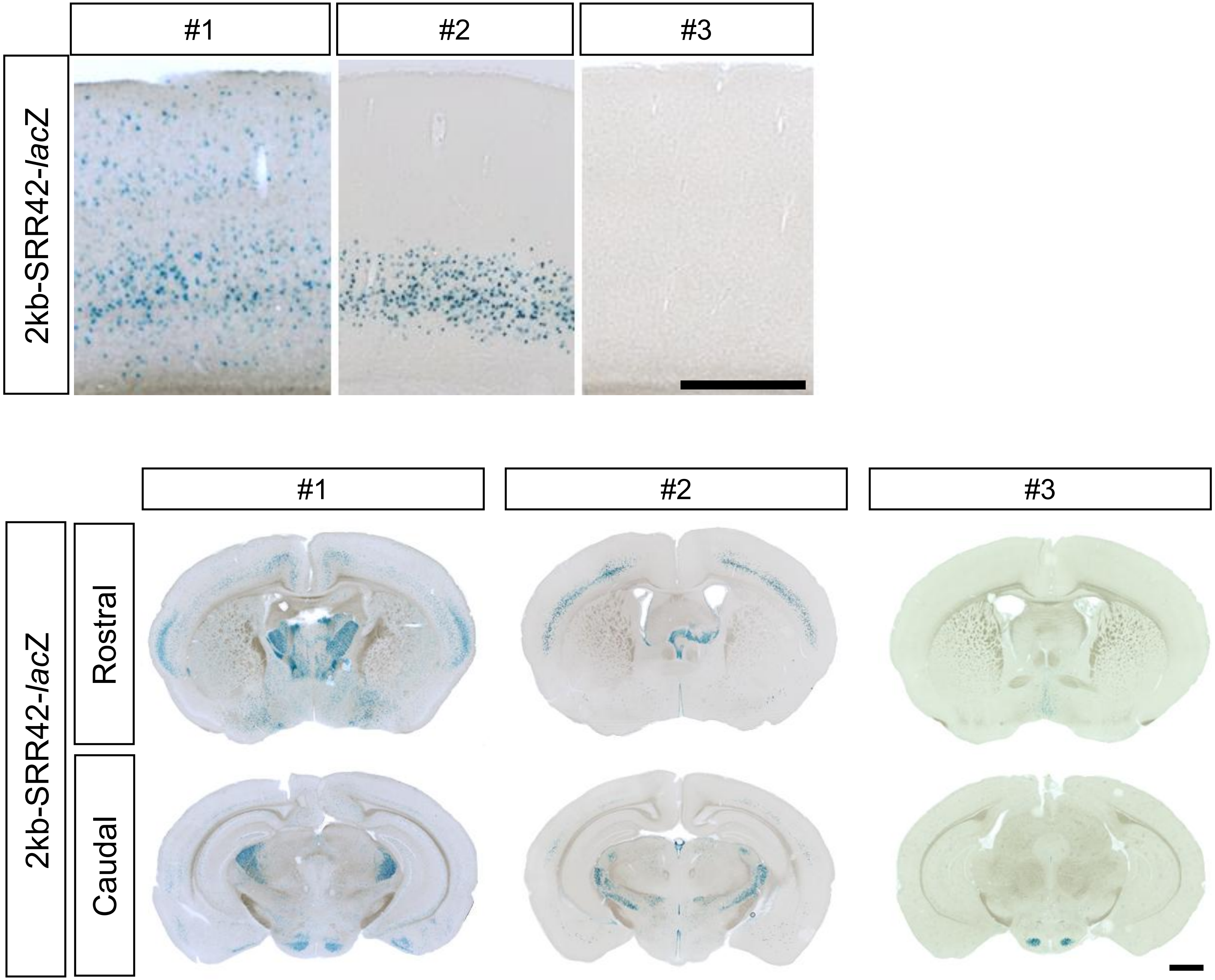

Figure S3

Figure S3. X-gal staining patterns in the brains of adult reporter mice.
Representative X-gal-stained images from founder lines #1 (left), #2 (middle), and #3 (right). The upper panels are coronal views of the cortex. Scale, 500 μm. The lower panels are entire brain views of rostral and caudal sides. Scale, 1 mm. X-gal staining was largely absent in line #3.

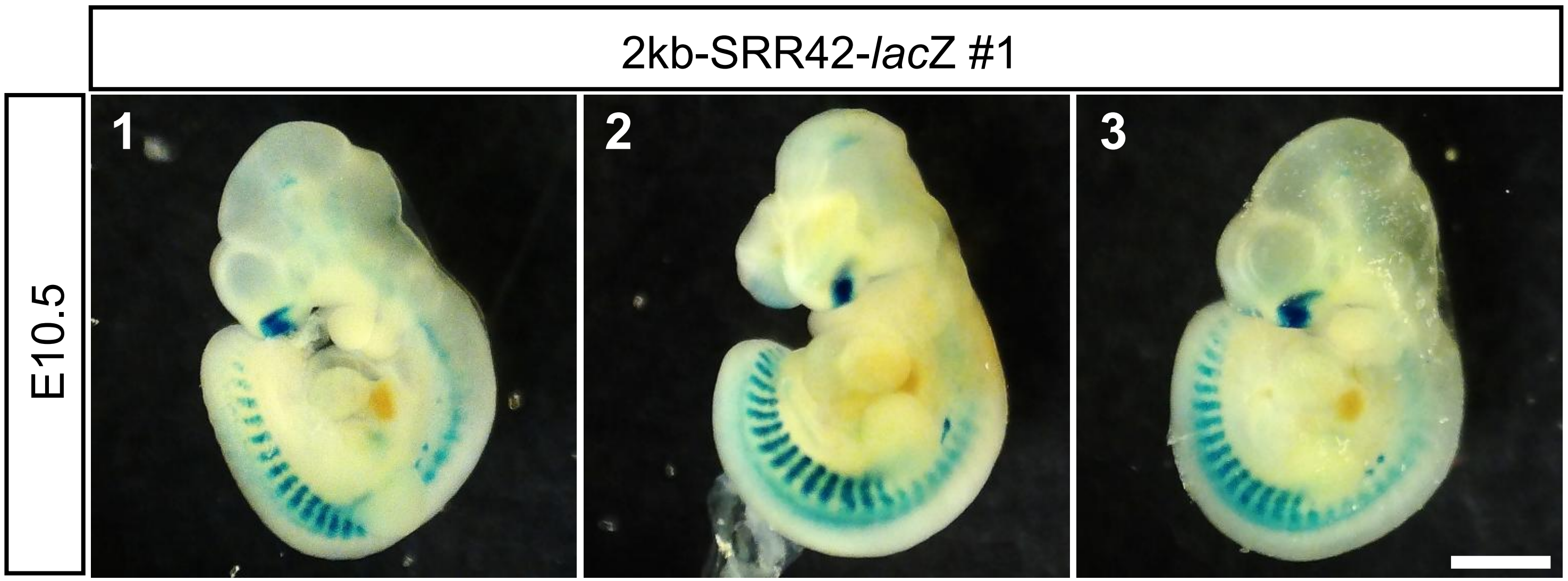

Figure S4

Figure S4. Reproducibility of X-gal staining patterns across independent transgenic embryos.
Whole-mount X-gal staining of E10.5 transgenic embryos carrying the 2-kb SRR42-LacZ reporter construct. Numbers 1–3 represent three independent embryos. Consistent staining was observed in the olfactory placode, neural tube, and dorsal root ganglia. Scale bar, 1 mm.

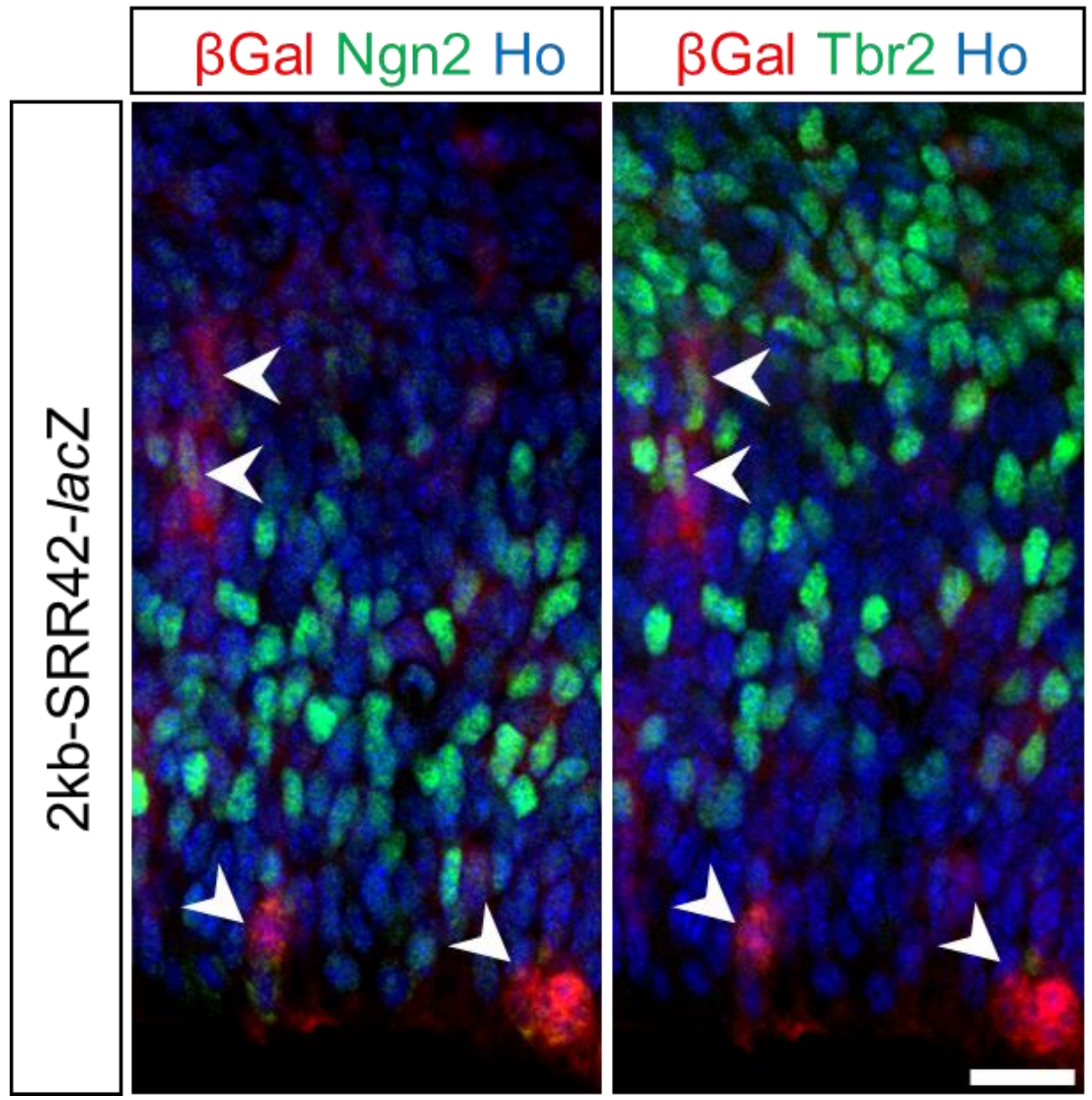

Figure S5

Figure S5. Co-staining of SRR42-LacZ reporter activity with neurogenic markers in the E14.5 cortex.
Representative images showing X-gal/LacZ reporter activity together with immunostaining for Neurogenin2 and Tbr2. LacZ-positive cells in the apical region showed limited overlap with Neurogenin2 or Tbr2, whereas more basally located LacZ-positive cells showed partial overlap with these markers. Scale bars, 20 μm.

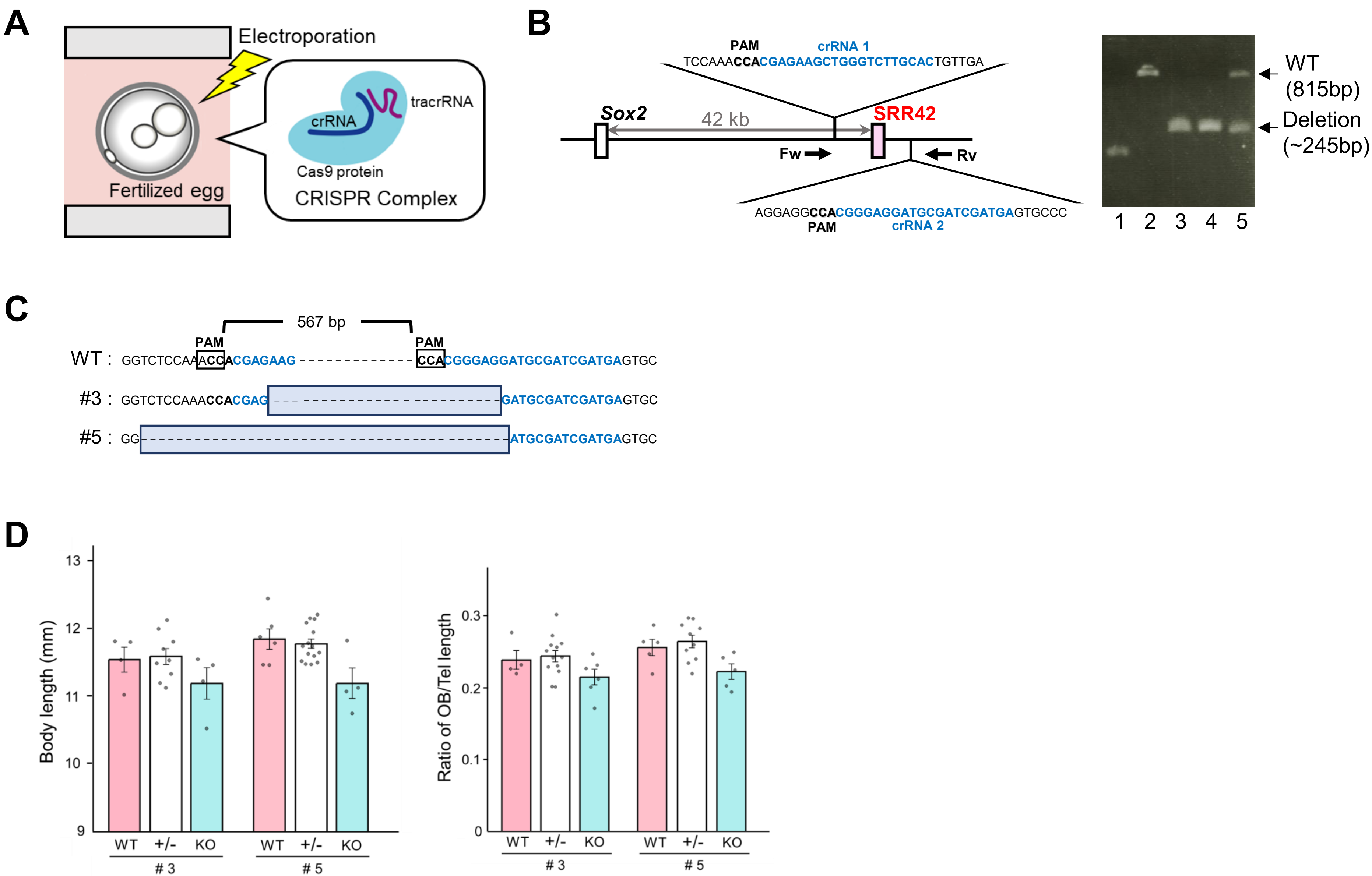
A
Electroporation
tracrRNA
crRNA
Cas9 protein
Fertilized egg
CRISPR Complex
B
PAM
crRNA 1
TCCAAACCACGAGAAGCTGGGTCTTGCACTGTTGA
Sox2
42 kb
SRR42
Fw
Rv
AGGAGGCCACGGGAGGATGCGATCGATGAGTGCCC
PAM
crRNA 2
WT
(815bp)
Deletion
(~245bp)
1 2 3 4 5
C
567 bp
PAM
PAM
WT : GGTCTCCAAACCACGAGAAG CCACGGGAGGATGCGATCGATGAGTGC
#3 : GGTCTCCAAACCACGAG GATGCGATCGATGAGTGC
#5 : GG ATGCGATCGATGAGTGC
D
Body length (mm)
13
12
11
10
9
WT +/- KO WT +/- KO
3
5
Ratio of OB/Tel length
0.3
0.2
0.1
0
WT +/- KO WT +/- KO
3
5

Figure S6

Figure S6. Generation and genotyping of SRR42-knockout (KO) mice.

A) Schematic illustration of CRISPR/Cas9-based generation of SRR42-KO mice. The in vitro fertilization zygotes were electroporated with Cas9 protein and sgRNAs following 6 h of gamete co-incubation.

B) Genomic structure of the SRR42 enhancer locus showing target sequences (blue), PAM sites, and primer regions for genotyping. PCR genotyping results are shown in the right panel.

C) Sequence alignment of the targeted genomic region in founder mice. Deleted regions are shown, with gRNA target sequences highlighted in blue. Both lines #3 and #5 showed complete SRR42 deletion.

D) Quantitative analysis of phenotypes in WT, heterozygous (+/–), and KO mice. Left: body length at E14.5. Right: ratio of olfactory bulb length to telencephalon length in P8 brains. Data represent mean ± SD for founder lines #3 and #5. The sequences of the primers used are listed in Supplementary Table 1.

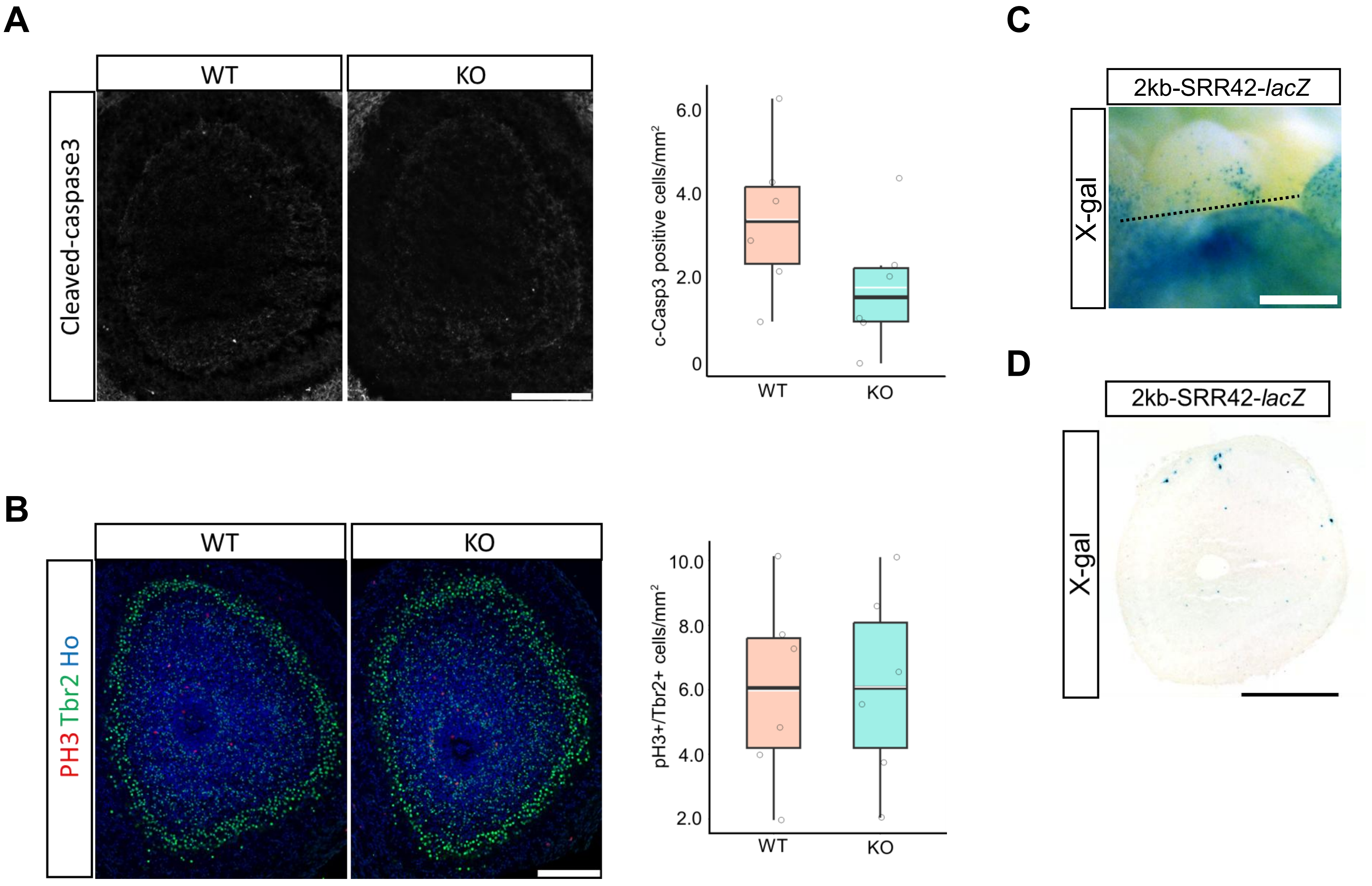

A
WT
KO
Cleaved-caspase3
c-Casp3 positive cells/mm²
6.0
4.0
2.0
0
WT
KO
B
WT
KO
PH3 Tbr2 Ho
pH3+/Tbr2+ cells/mm²
10.0
8.0
6.0
4.0
2.0
WT
KO
C
2kb-SRR42-lacZ
X-gal
D
2kb-SRR42-lacZ
X-gal

Figure S7

Figure S7. Normal cell survival and proliferation in the P0 olfactory bulb of KO mice.

A) Assessment of cell death in the P0 olfactory bulb (OB). Representative images of coronal sections of P0 OB immunostained for Cleaved-caspase3. Nuclei were counterstained with Hoechst. Scale bar, 200 μm. Box-and-whisker plots (right) show quantification, where black and white horizontal lines represent the median and mean, respectively.

B) Evaluation of cell proliferation in the P0 OB. Coronal sections of P0 OB double-immunostained for PH3 and Tbr2. Nuclei were counterstained with Hoechst. Scale bar, 200 μm. The accompanying box-and-whisker plots (right) quantify the number of PH3+/Tbr2+ cells; black and white bars indicate the median and mean, respectively.

C) Dorsal view of the P0 mouse OB. X-gal staining indicates regions of SRR42 activity. Dashed lines represent the planes of coronal sections shown in (D). Scale bar, 500 μm.

D) Spatial activity of SRR42 in the olfactory bulb. X-gal staining of P0 Tg mice identifying the anatomical regions where SRR42 is active within the OB. Scale bar: 500 μm.

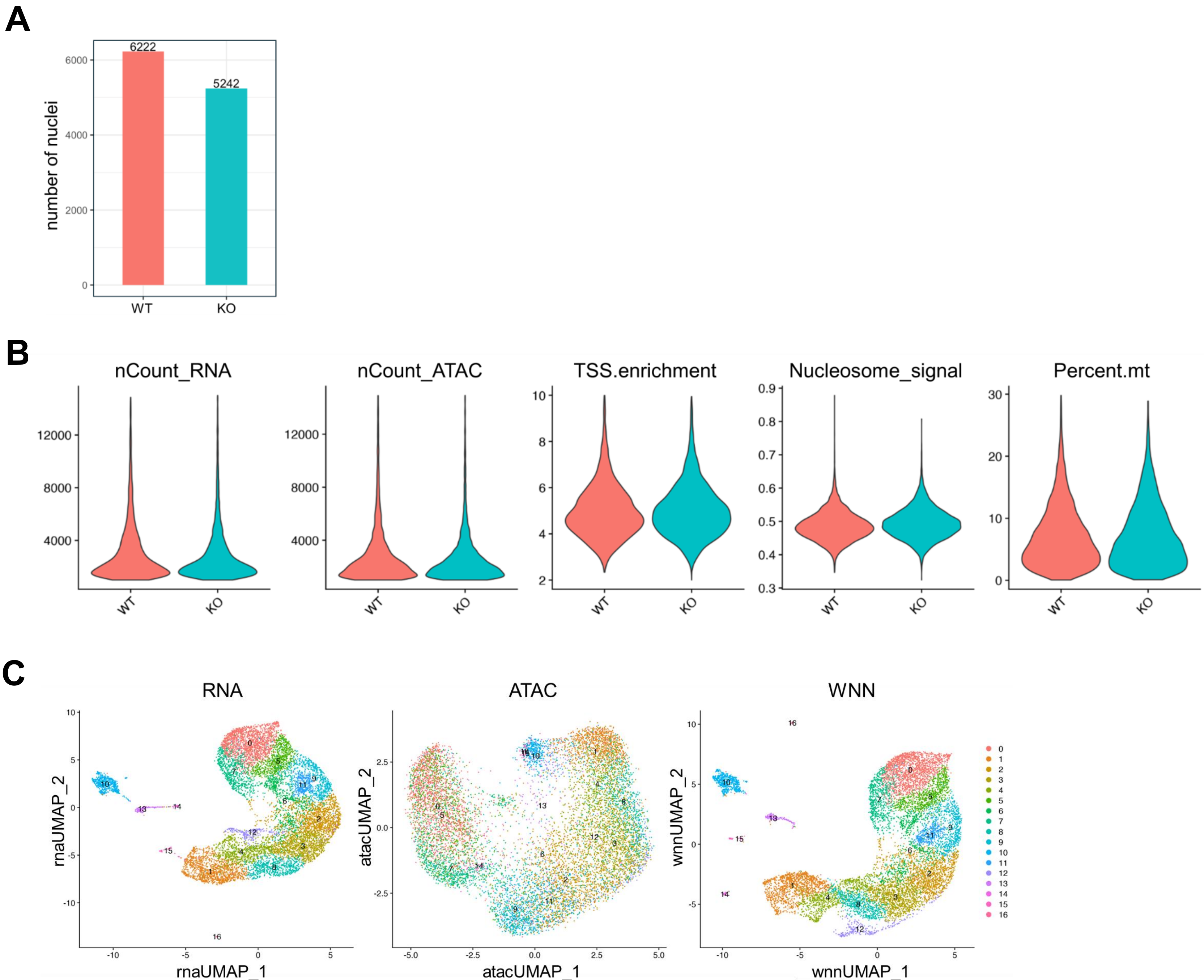
A
number of nuclei
6222
5242
WT
KO
B
nCount_RNA
nCount_ATAC
TSS.enrichment
Nucleosome_signal
Percent.mt
C
RNA
ATAC
WNN
rnaUMAP_1
rnaUMAP_2
atacUMAP_1
atacUMAP_2
wnnUMAP_1
wnnUMAP_2

Figure S8

Figure S8. The processing of single-cell multiome data of SRR42 WT and KO mice.

A) Bar graph displaying the number of nuclei obtained from the WT and KO mouse cortices.

B) Violin plots of quality control metrics including RNA counts, transcription start site (TSS) enrichment, nucleosome signal, and mitochondrial reads in nuclei of WT and KO mouse cortices.

C) UMAP projection of snRNA-Seq, snATAC-Seq, and integrated WNN analysis colored by WNN-annotated clusters.

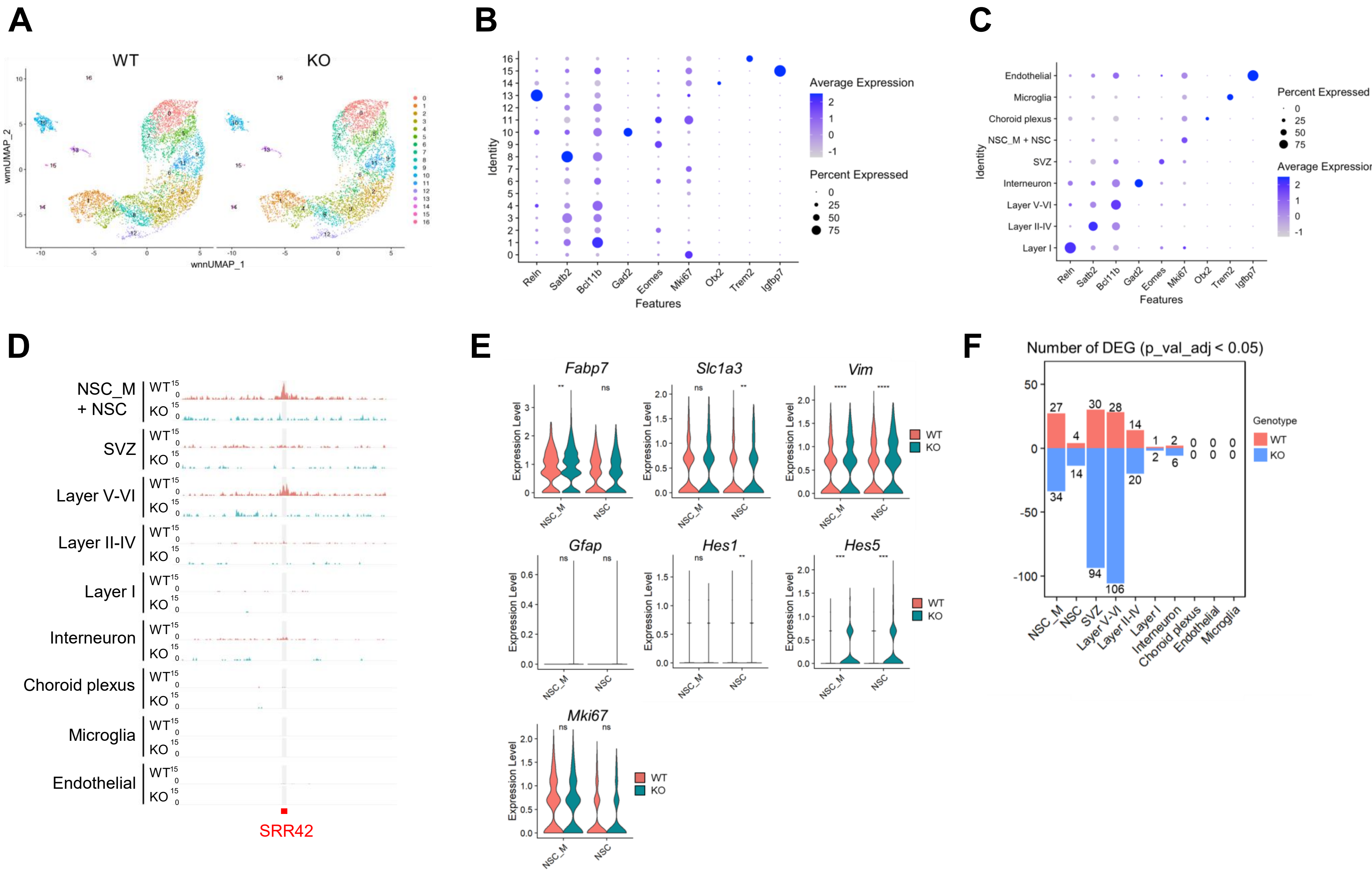

A
WT
KO
wnnUMAP_2
wnnUMAP_1
B
Average Expression
Percent Expressed
Identity
Features
Reln
Satb2
Bcl11b
Gad2
Eomes
Mki67
Otx2
Trem2
Igfbp7
C
Endothelial
Microglia
Choroid plexus
NSC_M + NSC
SVZ
Interneuron
Layer V-VI
Layer II-IV
Layer I
D
NSC_M + NSC
SVZ
Layer V-VI
Layer II-IV
Layer I
Interneuron
Choroid plexus
Microglia
Endothelial
SRR42
E
Fabp7
Slc1a3
Vim
Gfap
Hes1
Hes5
Mki67
Expression Level
NSC_M
NSC
F
Number of DEG (p_val_adj < 0.05)
Genotype
WT
KO

Figure S9

Figure S9. Marker gene expression and chromatin accessibility at the SRR42 region.

A) UMAP projection separated by genotypes in the WNN analysis colored by the identified clusters.
B) Dot plot showing scaled expression of selected marker genes across clusters.
C) Dot plot showing the scaled expression of selected marker genes across cell types.
D) Pseudo-bulk chromatin accessibility at the SRR42 region in WT and KO samples, based on pooled snATAC-Seq data from cortical cell types.
E) Violin plots showing expression of Fabp7, Slc1a3, Vim, Gfap, Neurog2, Hes1, Hes5, and Mki67 in NSC_M (WT: n = 942, KO: n = 882 cells) and NSC populations (WT: n = 818, KO: n = 694 cells), separated by genotype. ****$P < 0.0001$, ***$P < 0.001$, **$P < 0.01$; ns, not significant; Welch's *t*-test.
F) Bar plot displaying the number of DEGs identified in each cell type.

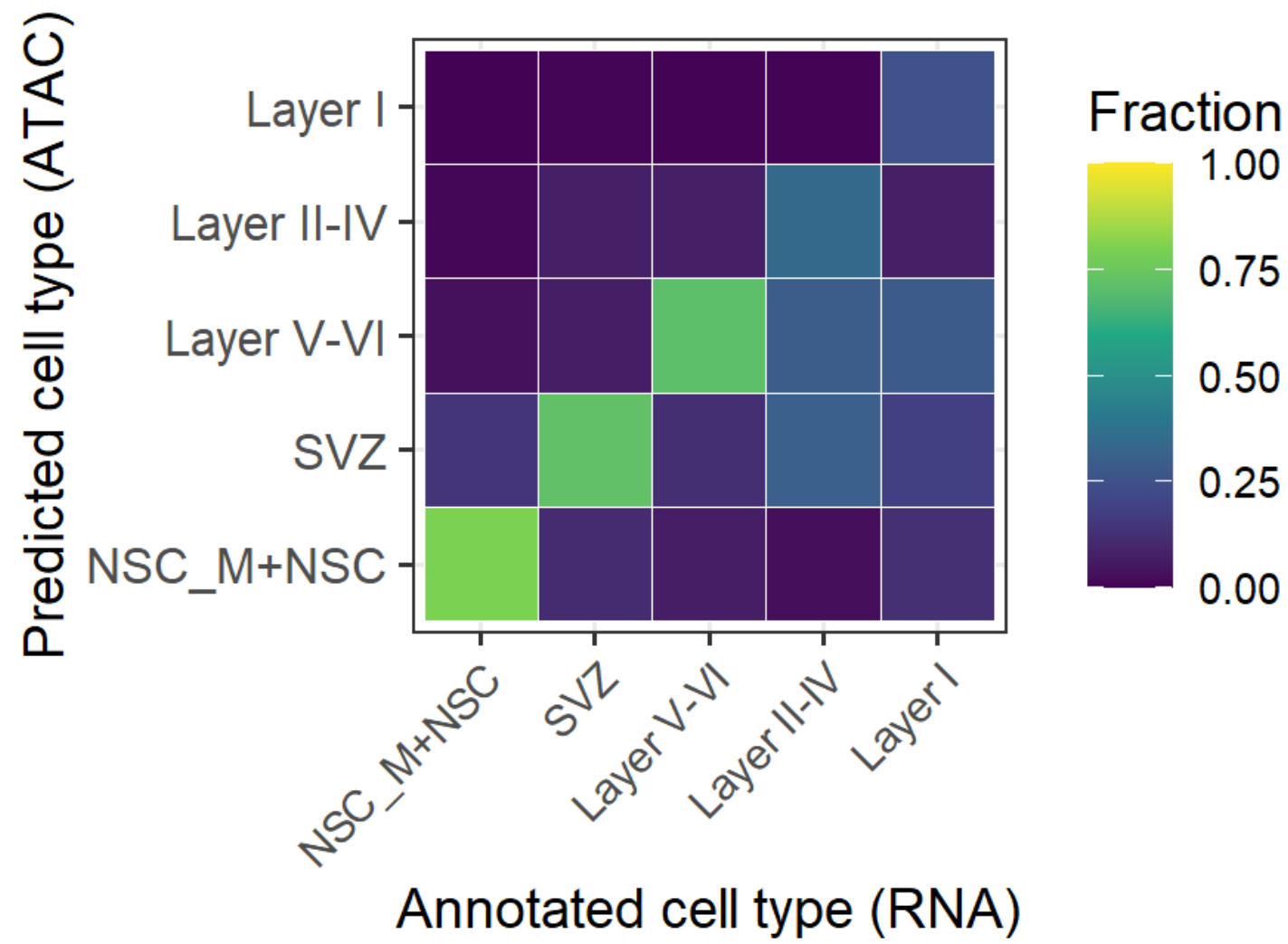

Predicted cell type (ATAC)
Layer I
Layer II-IV
Layer V-VI
SVZ
NSC_M+NSC
NSC_M+NSC
SVZ
Layer V-VI
Layer II-IV
Layer I
Annotated cell type (RNA)
Fraction
1.00
0.75
0.50
0.25
0.00

Figure S10

Figure S10. Consistent cell type identification between snRNA-seq and snATAC-seq data
Multimodal label transfer analysis between scRNA-seq and scATAC-seq datasets using Seurat. The heatmap illustrates the concordance between transcriptomic- and chromatin accessibility-based cell identity assignments. Color intensity represents the fraction of cells within each RNA-annotated cluster assigned to the predicted ATAC cell type.

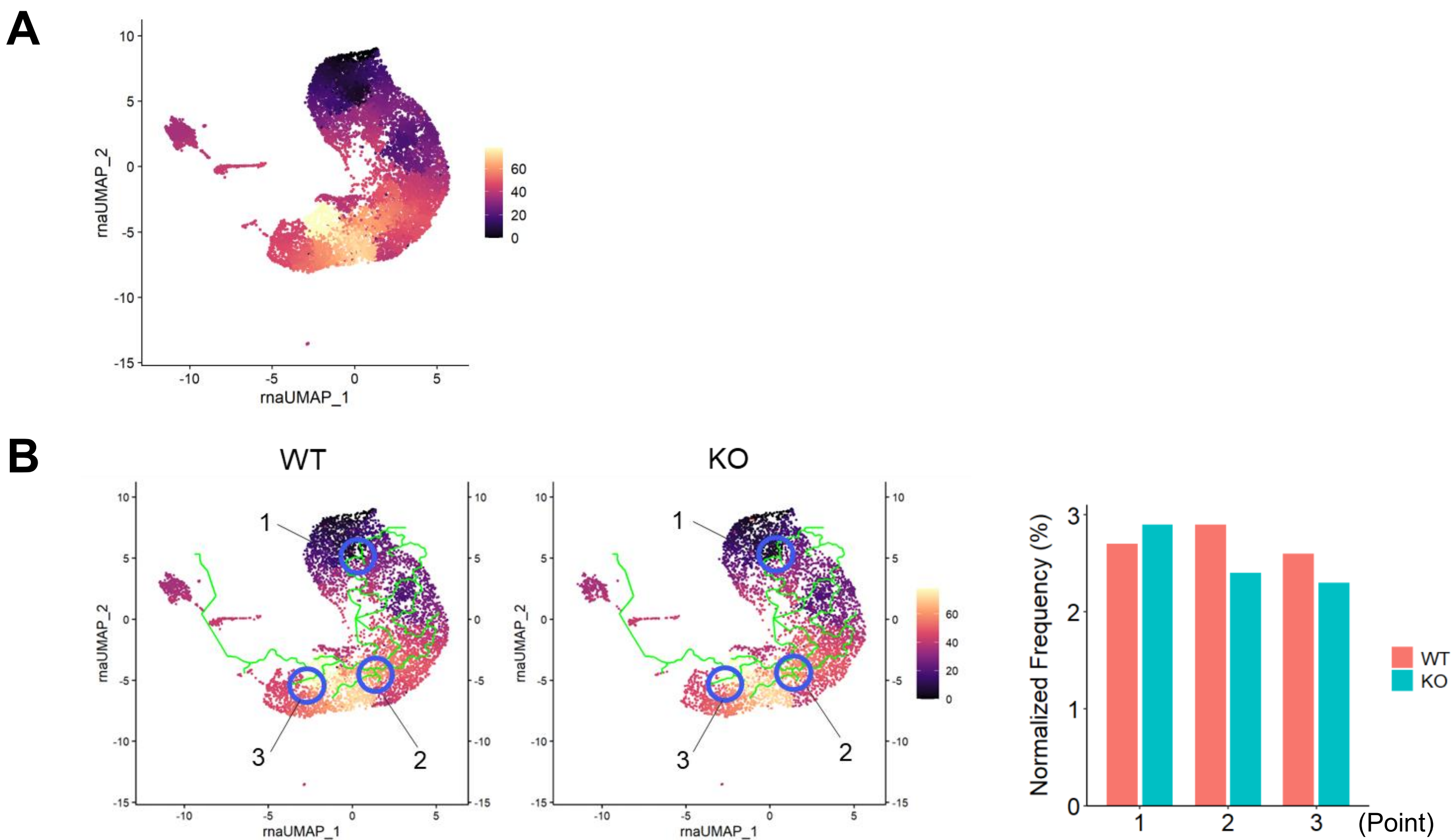
A
rnaUMAP_2
rnaUMAP_1
60
40
20
0
B
WT
KO
1
2
3
Normalized Frequency (%)
(Point)
WT
KO

Figure S11

Figure S11. Delayed differentiation trajectory in KO cells and cross-modality validation of cell states

A) UMAP plot colored by pseudotime. The differentiation transition was visualized using integrated scRNA-seq data from WT and KO E14.5 cortex. Darker shades (black) represent undifferentiated states, while lighter colors indicate progressive differentiation.

B) Developmental trajectories and cell proportions. Trajectories (green lines) are projected onto WT and KO UMAP plots. Circled regions (points 1–3) indicate specific developmental stages along the trajectory. The accompanying bar graphs (right) show the relative proportions of WT and KO cells at each indicated point.

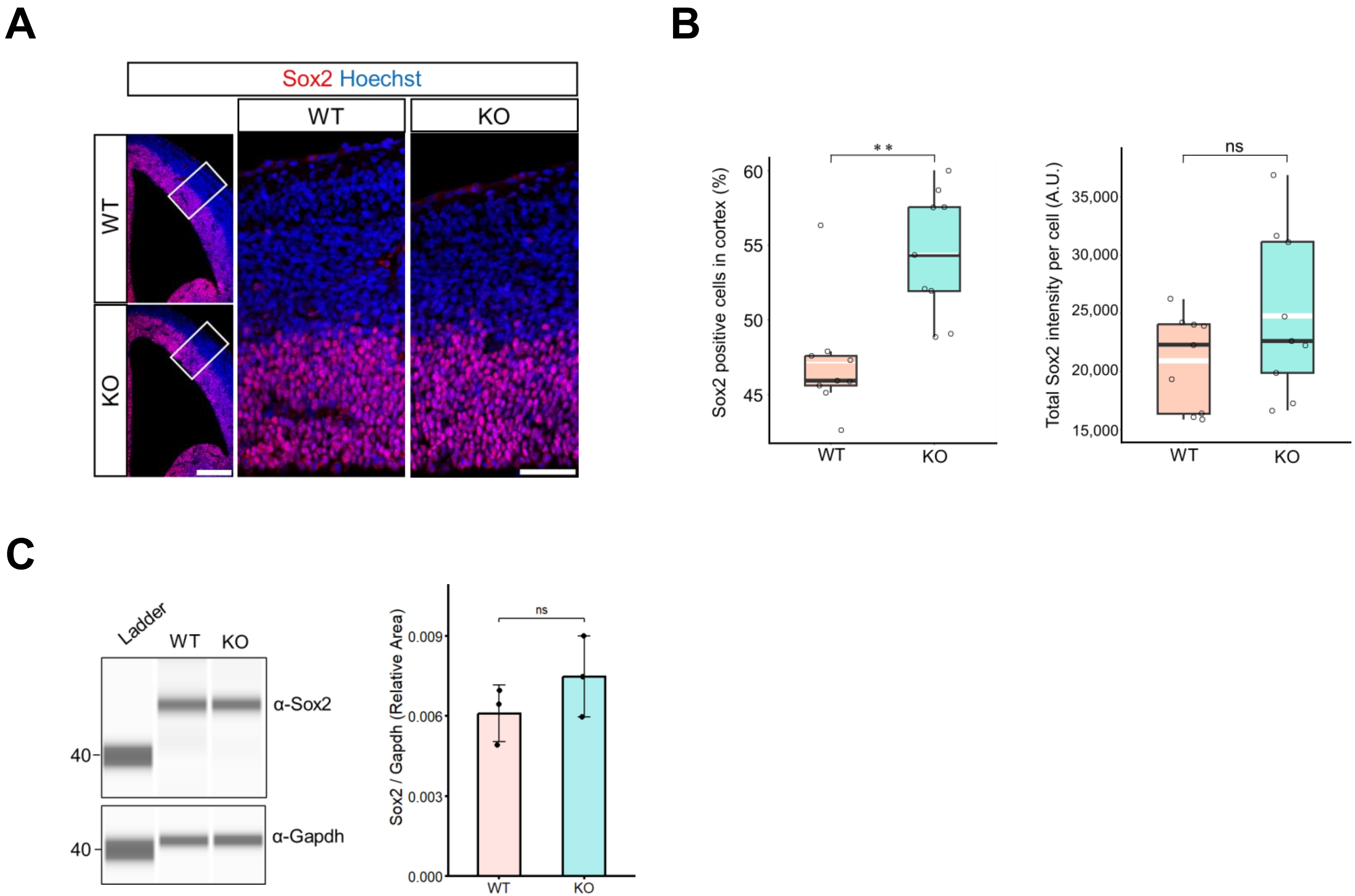
A
Sox2 Hoechst
WT
KO
WT
KO
B
Sox2 positive cells in cortex (%)
60
55
50
45
**
WT
KO
Total Sox2 intensity per cell (A.U.)
35,000
30,000
25,000
20,000
15,000
ns
WT
KO
C
Ladder
WT
KO
α-Sox2
α-Gapdh
40
40
Sox2 / Gapdh (Relative Area)
0.009
0.006
0.003
0.000
ns
WT
KO

Figure S12

Figure S12. Comparison of Sox2 protein expression levels in E14 cortex between WT and KO mice

A) Immunohistochemical analysis for Sox2 expression in the E14.5 cortex. Nuclei were counterstained with Hoechst. Scale bars: 200 μm (low-magnification overview) and 50 μm (high-magnification images).

B) Box-and-whisker plots show the quantification of Sox2-positive cells (left) and total Sox2 intensity normalized to cell number (right) in cortex. In the box plots, black and white lines represent the median and mean, respectively. Statistical significance was determined by Welch's *t*-test (**$P < 0.01$; ns, not significant; $n = 9$ sections from $N = 3$ mice per genotype).

C) Analysis of Sox2 protein expression in the E14.5 mouse cortex. (Left) Representative immunoblot image for Sox2 and Gapdh protein levels in the E14.5 cortex of WT and KO mice acquired using Simple Western system . (Right) Quantification of Sox2 protein levels normalized to Gapdh ($N = 3$ per genotype). *P*-values were calculated using Type II ANOVA (car package). "ns" indicates no statistical significance ($P > 0.05$).

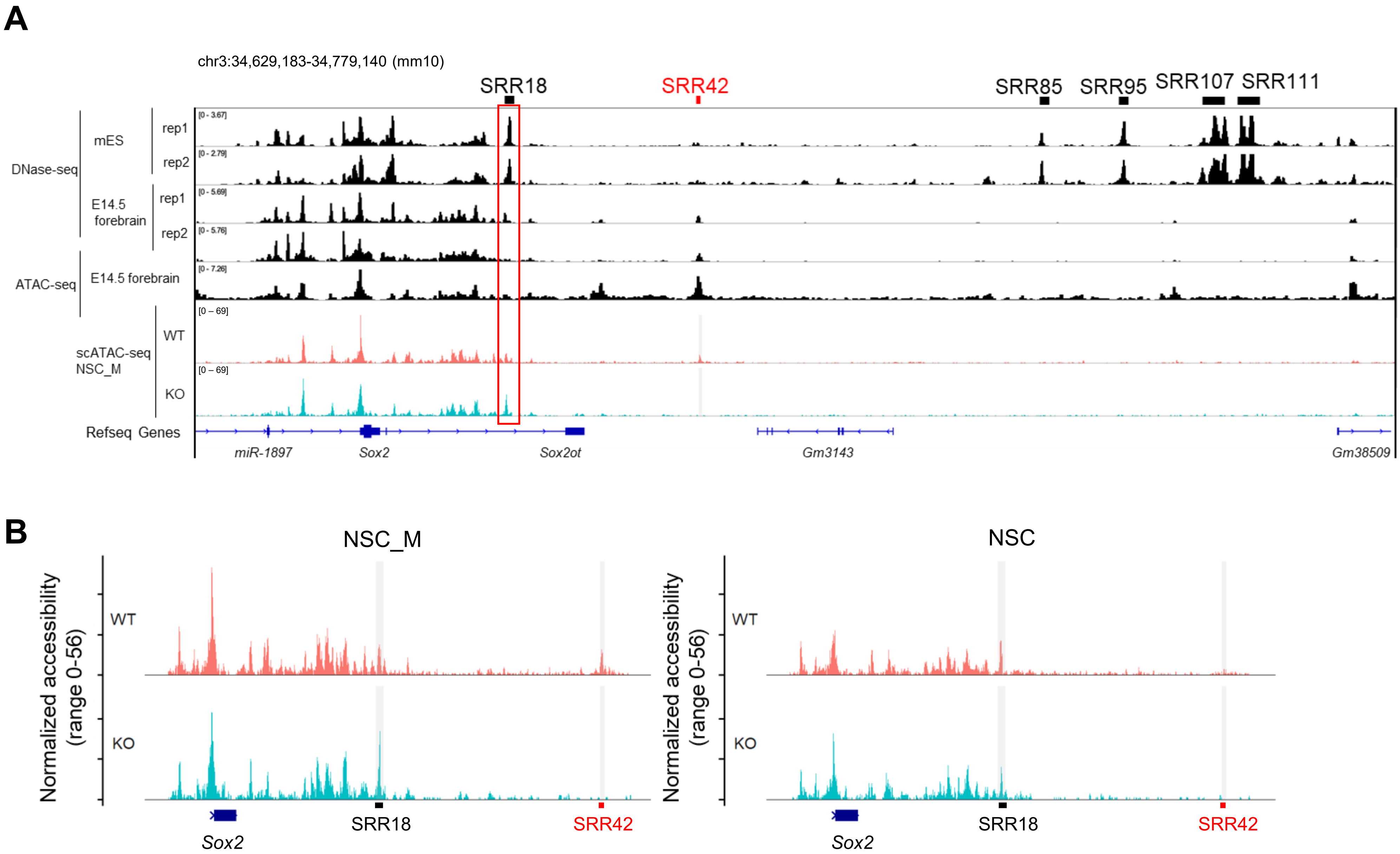
A
chr3:34,629,183-34,779,140 (mm10)
SRR18
SRR42
SRR85
SRR95
SRR107
SRR111
DNase-seq
mES
rep1
rep2
E14.5 forebrain
rep1
rep2
ATAC-seq
E14.5 forebrain
scATAC-seq NSC_M
WT
KO
[0 – 69]
[0 – 69]
Refseq Genes
miR-1897
Sox2
Sox2ot
Gm3143
Gm38509
B
NSC_M
NSC
Normalized accessibility (range 0-56)
WT
KO
Sox2
SRR18
SRR42

Figure S13

Figure S13. Chromatin accessibility across Sox2 regulatory regions in SRR42-KO cells.

A) Chromatin accessibility across SRR loci. Genome browser tracks showing DNase-seq and ATAC-seq profiles in mESCs and E14.5 forebrain at SRR18, 42, 85, 95, 107, and 111, compared with scATAC-seq data of normalized signal from WT and KO NSC_M. While no differences were observed at SRR85, 95, 107, or 111 in the KO, a distinct peak was identified at SRR18.

B) Cell-type-specific scATAC-seq profiles. Genome browser views (chr3:34,645,405-34,702,461; mm10) of scATAC-seq data in NSC_M and general NSC clusters. The SRR18 and SRR42 peaks are highlighted with a gray background. The SRR18 peak alteration observed in NSC_M was not detected in the broader NSC population.

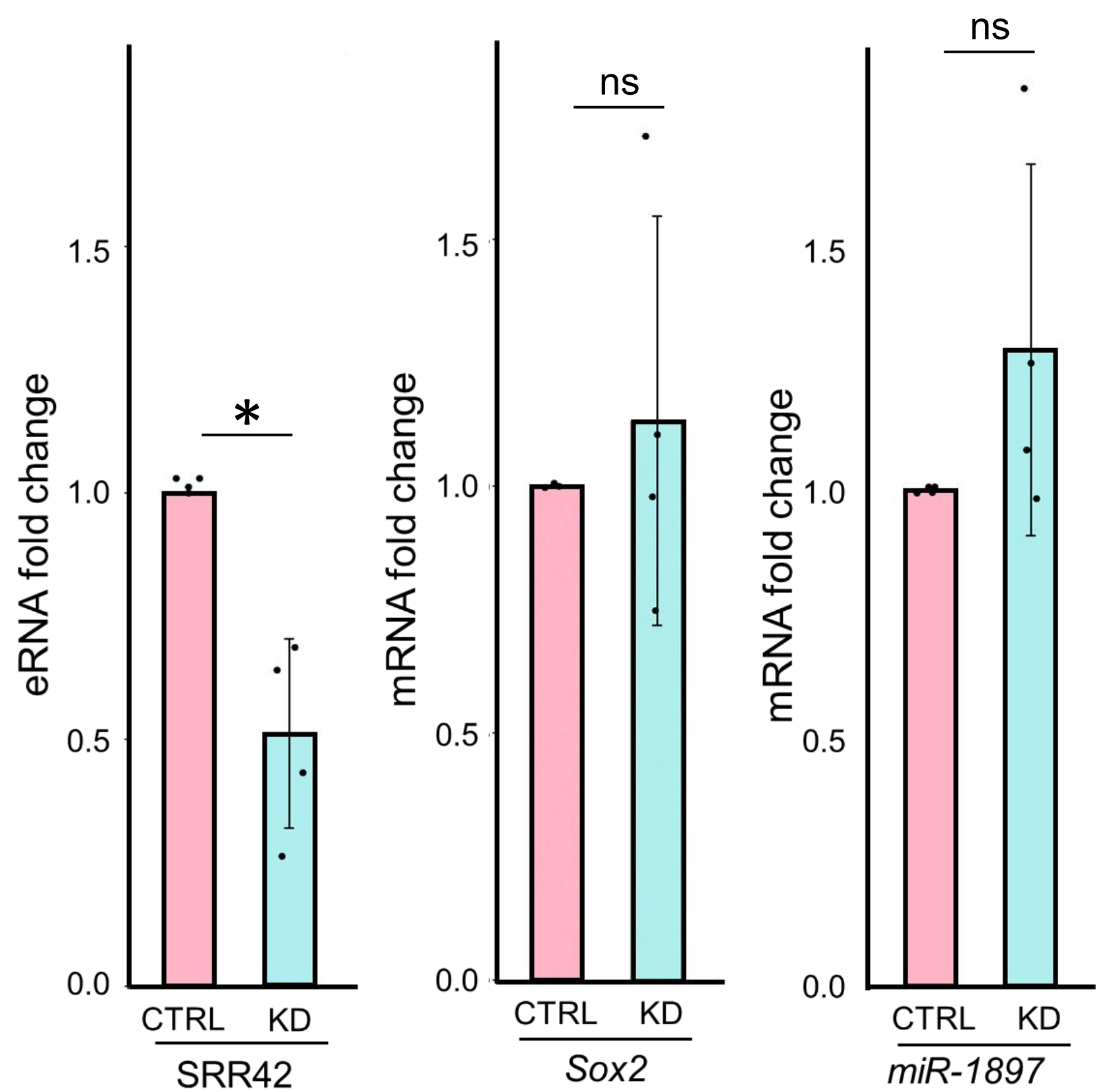
eRNA fold change
1.5
1.0
0.5
0.0
*
CTRL
KD
SRR42
mRNA fold change
ns
Sox2
mRNA fold change
ns
miR-1897

Figure S14

Figure S14. CasRx knockdown of SRR42 eRNA without affecting neighboring genes.

Bar graphs showing the expression levels of the target SRR42 eRNA (left) and neighboring genes, Sox2 (middle) and miR-1897 (right) following Rfx-Cas13d-mediated knockdown in mouse neuronal cells from E14.5 cortex. Relative expression was determined by RT-qPCR. Expression levels were normalized to Gapdh and calculated using the $2^{-\Delta\Delta Ct}$ method. n = 4 biologically independent experiments. Data are presented as the mean ± SD. Statistical significance was determined by a two-tailed paired t-test performed on ΔCt values. P-values for SRR42 ($P < 0.05$), Sox2 ($P = 0.6857$) and miR-1897 ($P = 0.2026$) are indicated above the respective bars.

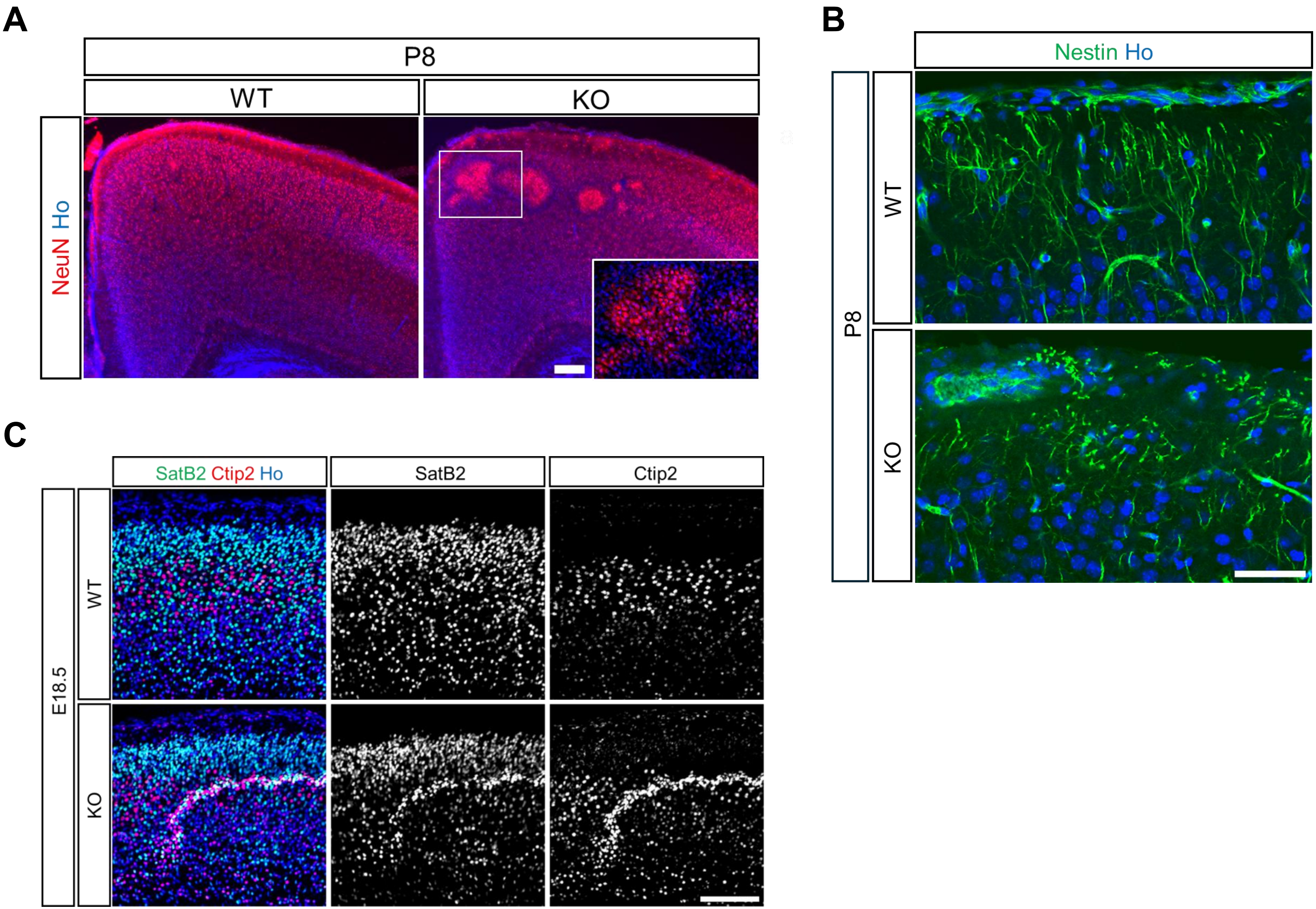
A
P8
WT
KO
NeuN Ho
B
Nestin Ho
P8
WT
KO
C
SatB2 Ctip2 Ho
SatB2
Ctip2
E18.5
WT
KO

Figure S15

Figure S15. Loss of the SRR42 enhancer causes cortical dysplasia in mice.

A) Immunostaining of P8 KO cortex showing dysplastic regions labeled with NeuN (red) and Hoechst (blue). Scale, 200 μm.
B) Higher-magnification image of the cortical plate stained with Nestin (green) and Hoechst (blue). Scale, 50 μm.
C) Immunostaining of an E18.5 KO cortex showing heterotopic cell clusters. Satb2 (green), Ctip2 (red), and nuclei (Hoechst, blue) are shown. Scale, 100 μm.
Sources and dilutions of the antibodies used, along with Hoechst staining details, are listed in Supplementary Table 2.

Table S1. List of primers used in the study

Primer Name	Sequence
**Transgene construction**	**5' ----> 3'**
SRR42_transgene_2k_Fw	CACCcaataacgccactttaacaaac
SRR42_transgene_2k_Rv	ccaggccagtcaaggctatcttg
**crRNA for deficient mice**	**5' ----> 3'**
SRR42_5prime	GTGCAAGACCCAGCTTCTCG TGG
SRR42_3prime	TCATCGATCGCATCCTCCCG TGG
**Genotyping PCR pimer**	**5' ----> 3'**
SRR42_del_Fw	CCCTTTGGTCATCACCTCTC
SRR42_del_Rv	AAGCCAGAACAACGTAGACC
**Target sequence of eRNA knockdown**	**5' ----> 3'**
SRR42_target_1	GCGCGTTACATGCTTGCAAATCGCCCCACC
SRR42_target_2	CTCCTCCTCTGCAGCCATGGTCACTTGTAC
**qPCR primer**	**5' ----> 3'**
SRR42_eRNA_3_Fw	ATGGTCACTTGTACCACGAG
SRR42_eRNA_3_Rv	AGGCAGGATCTTTCCTCTTCC
SRR42_eRNA_4_Fw	GCAAAAATTTTCAGCCTCACGGG
SRR42_eRNA_4_Rv	ACGGACTTCTCTATGTGGCCTCG
mSox2_Fw	GCGGAGTGGAAACTT TTGTCC
mSox2_Rv	GGGAAGCGTGTACTTATCCTCT
mSox2ot_Fw	CACGGGACGAAAGGATGAGAG
mSox2ot_Rv	TATGGATAAAGTCGGCGACG
mmu-miR1897_Fw	ATTCTCCGTGCTTTGGATGG
mmu-miR1897_Rv	TGGATCACTCCTCACCGGAC
mGAPDH_Fw	CATGGCCTTCCGTGTTCCTA
mGAPDH_Rv	GCCTGCTTCACCACCTTCTT

Table S2. List of antibodies and plasmids used in the study.

Antibodies for immunostaining	SOURCE	IDENTIFIER	DILUTION
Anti-β-galactosidase	MBL	Cat# M203-3	1/200
Anti-Sox2	R&D Systems	Cat# MAB2018	1/200
Anti-SatB2	Abcam	Cat# ab92446	1/200
Anti-BrdU	Abcam	Cat# ab6326	1/500
Anti-Ctip2	Abcam	Cat# ab18465	1/200
Anti-NeuN	Millipore	Cat# MAB377	1/200
Anti-Phospho-Histone H3 (pH3)	Cell Signaling Technology	Cat# 9706	1/100
Anti-Tbr2	Millipore	Cat# AB15894	1/100
Anti-Ds-Red	Nittobo Medical	Cat# MSFR101410	1/200
Anti-Nestin	Millipore	Cat# MAB353	1/200
Anti-Cleaved Caspase-3	Cell Signaling Technology	Cat# 9661	1/250
Anti-Neurogenin-2	Millipore	Cat# MABN1814	1/200

Antibodies for simple western	SOURCE	IDENTIFIER	DILUTION
Anti-Sox2	R&D Systems	Cat# MAB2018	1/250
Anti-GAPDH	Cell Signaling Technology	Cat# 2118	1/50

Plasmids	SOURCE	IDENTIFIER
Hsp68-LacZ-Gateway	Addgene	Plasmid #37843
pENTR/D-TOPO vector	Thermo Scientific	K240020SP
Hsp68-LacZ-SRR42_transgene_2k	This study	N/A
pCAGGS-mRFP	Kind gift from Dr. Ayako Y. Murayama	N/A
pCAGGS-mEGFP	Kind gift from Dr. Ayako Y. Murayama	N/A
AAV-EFS-NLS-CasRx-U6-DR-sgRNA_LacZ-DR	Addgene	Plasmid #154003
AAV-EFS-NLS-CasRx-U6-DR-sgRNA_SRR42_1-2-DR	This study	N/A
